# Supplementary material for: A Systematic Study of the In Vitro Pharmacokinetics and Estimated Human In Vivo Clearance of Indole and Indazole-3-Carboxamide Synthetic Cannabinoid Receptor Agonists Detected on the Illicit Drug Market
Source: Molecules. 2021 Mar 5;26(5):1396. doi: 10.3390/molecules26051396 (PMC7961380; doi:10.3390/molecules26051396)
Supplement: Supplementary file 1 [file molecules-26-01396-s001.pdf]

A systematic study of the *in vitro* pharmacokinetics and estimated human *in vivo* clearance of indole and indazole-3-carboxamide synthetic cannabinoid receptor agonists detected on the illicit drug market

Andrew M. Brandon[1], Lysbeth Antonides[1], Jennifer Riley[2], Ola Epemolu[2], Denise A. McKeown[3], Kevin D. Read[2*], Craig McKenzie[1*]

[1] Leverhulme Research Centre for Forensic Science, School of Science and Engineering, University of Dundee, Dundee, UK; [2] Drug Discovery Unit, School of Life Sciences, University of Dundee, Dundee, UK; [3] Forensic Medicine and Science, School of Medicine, Dentistry and Nursing, College of Medical, Veterinary and Life Sciences, University of Glasgow, Glasgow, UK

Supplementary Information

Section S1: Mass spectrometry multiple reaction monitoring transitions

Section S2: *In silico* log P prediction using multiple software packages

Section S3: Equations and literature chromatographic hydrophobicity index data used in determining CHI Log D values

Section S4: Equations used for *in vitro* intrinsic clearance calculation and human *in vivo* clearance estimation

Section S5: Plasma stability data

Section S6: Intrinsic clearance data – human liver microsome incubations

Section S7: Intrinsic clearance data – human hepatocyte incubations

Section S1

Mass spectrometry multiple reaction monitoring transitions

Multiple reaction monitoring (MRM) transitions monitored for each SCRA in analysis by mass spectrometry are provided in Table S1. Transitions used were determined by tuning the instrument to each compound.

Table S1 Multiple reaction monitoring (MRM) transitions for tested synthetic cannabinoid receptor agonists.

Compound	Transition (Da)
(*S*)-AMB-FUBINACA (**1**)	384.01 -> 108.86
(*R*)-AMB-FUBINACA (**1**)	384.01 -> 108.83
(*S*)-5F-MDMB-PINACA (**2**)	378.09 -> 318.07
(*R*)-5F-MDMB-PINACA (**2**)	378.07 -> 232.90
(*S*)-5F-MDMB-PICA (**3**)	377.09 -> 231.94
(*R*)-5F-MDMB-PICA (**3**)	377.12 -> 231.94
(*S*)-4F-MDMB-BINACA (**4**)	364.07 -> 218.93
(*R*)-4F-MDMB-BINACA (**4**)	364.08 -> 218.95
(*S*)-MDMB-4en-PINACA (**5**)	358.07 -> 212.90
(*R*)-MDMB-4en-PINACA (**5**)	358.05 -> 212.90
(*S*)-AB-FUBINACA (**8**)	369.03 -> 108.83
(*R*)-AB-FUBINACA (**8**)	369.03 -> 108.80
(*S*)-AMB-4en-PICA (**7**)	343.00 -> 211.91
(*R*)-AMB-4en-PICA (**7**)	343.11 -> 211.95
(*S*)-MDMB-FUBINACA (**6**)	398.05 -> 108.79
(*R*)-MDMB-FUBINACA (**6**)	398.06 -> 108.82
(*S*)-AB-CHMINACA (**11**)	357.03 -> 340.03
(*R*)-AB-CHMINACA (**11**)	357.07 -> 240.97
(*S*)-AMB-CHMICA (**9**)	371.11 -> 239.97
(*R*)-AMB-CHMICA (**9**)	371.13 -> 239.98
(*S*)-5F-AMB-PINACA (**10**)	364.07 -> 232.91
(*R*)-5F-AMB-PINACA (**10**)	364.08 -> 232.90
(*S*)-MDMB-4en-PICA (**12**)	357.12 -> 211.92
(*R*)-MDMB-4en-PICA (**12**)	356.98 -> 211.96

Section S2

In silico log P prediction using multiple software packages

Log P values predicted using various pharmacokinetic prediction tools are provided in Table S2. Software packages used were SwissADME (Swiss Institute of Bioinformatics, Lausanne, Switzerland; online), Gastroplus (SimulationPlus, Lancaster, CA, USA; MedChem Designer version 4.5), MoKa (Molecular Discovery, Borehamwood, UK; version 3.0), Canvas (Schrödinger, LLC, New York, NY, USA; version 3.6) and XlogP (Institute of Physical Chemistry, Peking University).

Table S2 *In silico* log P predictions using various software packages.

Compound	SwissADME	Gastroplus	MoKa	Canvas	XlogP
5F-MDMB-PICA (**3**)	3.98	4.10	4.20	4.12	4.90
AMB-CHMICA (**9**)	3.84	4.48	4.30	4.53	5.51
MDMB-FUBINACA (**6**)	3.83	4.19	4.10	4.08	4.24
MDMB-4en-PICA (**12**)	3.77	3.98	4.20	4.01	4.98
5F-MDMB-PINACA (**2**)	3.63	3.76	3.90	3.79	3.79
AMB-4en-PICA (**7**)	3.53	3.50	3.90	3.74	4.83
AMB-FUBINACA (**1**)	3.50	3.75	3.80	3.81	4.09
MDMB-4en-PINACA (**5**)	3.41	3.61	4.00	3.69	3.87
5F-AMB-PINACA (**10**)	3.41	3.31	3.60	3.52	3.64
4F-MDMB-BINACA (**4**)	3.39	3.38	3.40	3.33	3.34
AB-CHMINACA (**11**)	2.91	3.12	3.10	3.32	3.55
AB-FUBINACA (**8**)	2.80	2.66	2.90	2.92	3.24
5F-AB-P7AICA	2.45	1.57	2.10	1.91	2.74

Section S3

Equations and literature chromatographic hydrophobicity index data used in determining CHI Log D values

Equations used in determining CHI Log D values are provided below. Literature CHI data (CHI_0) for calibration standards is provided in Table S3. The plot of experimental retention factor (k) against CHI_0 used in Equation 4 is displayed in Figure S1.

Calculation of retention factor (k)

$$k = \frac{(t_r - t_0)}{t_0} \qquad \textit{Eqn.3}$$

Where t_r is the retention time of the analyte and t_0 is the retention time of an unretained compound (uracil)

Calculation of chromatographic hydrophobicity index (CHI)

$$\mathrm{CHI} = (k \text{ x slope}) - \text{intercept} \qquad \textit{Eqn.4}$$

Where k is the retention factor of the analyte. Slope and intercept are taken from the line equation of the plot of calibration compound retention factor against literature CHI (Figure S1)

Calculation of CHI Log D [1]

$$\text{CHI Log D} = (0.0525 \text{ x CHI}) - 1.467 \qquad \textit{Eqn.5}$$

Where CHI is the chromatographic hydrophobicity index obtained using Equation 4

Table S3 Literature chromatographic hydrophobicity index (CHI) values (CHI_0) [2]

Compound	CHI_0 (pH 7.4)
Theophylline	18.4
Phenyltetrazole	23.6
Benzimidazole	34.3
Colchicine	43.9
Phenyltheophylline	51.7
Acetophenone	64.1
Indole	72.1
Propiophenone	77.4

Figure S1 Plot of retention factor (k) against CHI_0

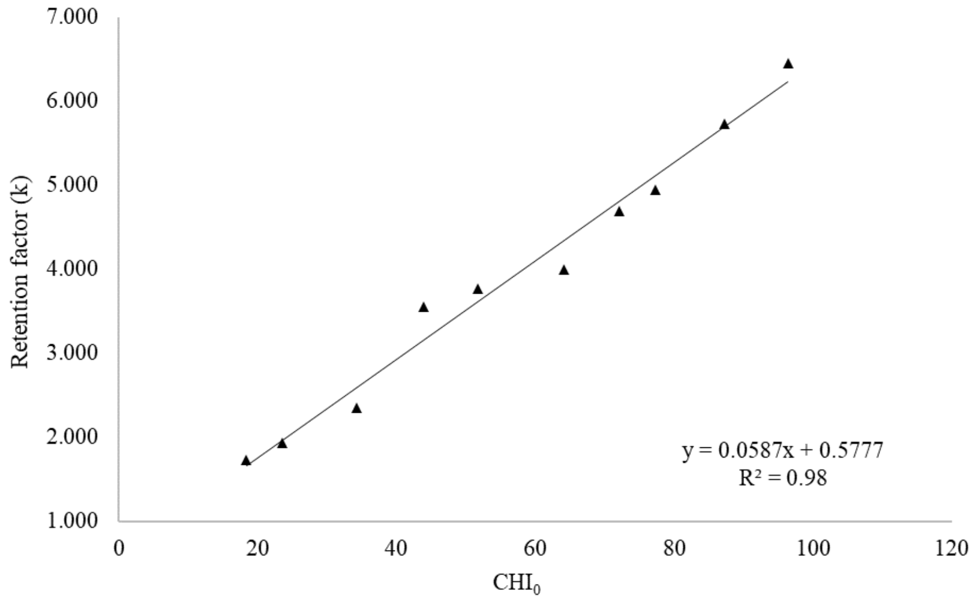

References

1. Young, R.G.; Green, D.V.S.; Luscombe, C.N., Hill, A.P. Getting physical in drug discovery II: the impact of chromatographic hydrophobicity measurements and aromaticity. *Drug Discov. Today*. **2011**, *16*, 822-830. doi: 10.1016/j.drudis.2011.06.001.

2. Camurri, G.; Zaramella, A. High-throughput liquid chromatography/mass spectrometry method for the determination of the chromatographic hydrophobicity index. *Anal. Chem.* **2001**, *73*, 3716-3722. doi: 10.1021/ac001388j.

Section S4

Equations used for *in vitro* intrinsic clearance calculation and human *in vivo* clearance estimation

The equations used for *in vitro* intrinsic clearance calculation and human *in vivo* clearance estimation were as described by Baranczewski et al. (2006) [3], Obach et al. (1997) [4] and Rane, Wilkinson & Shand (1977) [5].

<u>Microsomal clearance (HLM incubations)</u> in mL min^{-1} mg microsomal $protein^{-1}$ [3]

$$CL_{int\ micr} = k\ x\ \frac{V}{P} \qquad \textit{Eqn.6}$$

Where: k = rate constant (min^{-1}) = $0.693/t_{1/2}$; V = incubation volume (mL); P = microsomal protein (mg)

<u>Intrinsic clearance (whole-liver dimensions) (HLM incubations)</u> in mL min^{-1} kg^{-1} [3]

$$CL_{int} = k\ x\ \frac{V}{P}\ x\ \frac{[Liver](g)}{[BW](kg)}\ x\ SF \qquad \textit{Eqn. 7}$$

Where: [Liver] = weight of liver, 1799 g [6]; [BW] = body weight, 70 kg; SF = scaling factor, 52.5 mg g microsomal $protein^{-1}$ [7]

<u>Intrinsic clearance (whole-liver dimensions) (HHep incubations)</u> in mL min^{-1} kg^{-1} [4]

$$CL_{H\ int} = k\ x\ \frac{V}{[hepatocytes]}\ x\ hepatocytes/g\ liver\ x\ \frac{[Liver](g)}{[BW](kg)} \qquad \textit{Eqn. 8}$$

Where: hepatocytes/g liver = 107×10^6 cells/g liver [8]

<u>Hepatic clearance</u> in mL min^{-1} kg^{-1} [3]

$$CL_H = \frac{Q\ x\ fu\ x\ CL_{int}}{Q + (fu\ x\ CL_{int})} \qquad \textit{Eqn. 9}$$

Where: Q = hepatic blood flow, 21 mL min^{-1} kg^{-1} [9]; fu = fraction unbound (plasma); CL_{int} = intrinsic clearance

<u>Estimated human hepatic extraction ratio</u> [5]

$$E_H = \frac{CL_H}{Q} \qquad \textit{Eqn. 10}$$

References

3. Baranczewski, P.; Stanczak, A.; Sundberg, K.; Svensson, R.; Wallin, A.; Jansson, J.; Garberg, P.; Postlind, H. Introduction to in vitro estimation of metabolic stability and drug interactions of new chemical entities in drug discovery and development. *Pharmacol. Rep.* **2006**, *58,* 453-472.

4. Obach, R.S.; Baxter, J.G.; Liston, T.E.; Silber, B.M.; Jones, B.C.; MacIntyre, F.; Rance, D.J.; Wastall, P. The prediction of human pharmacokinetic parameters from preclinical and in vitro metabolism data. *J. Pharmacol. Exp. Ther.* **1997**, *283(1),* 46-58.

5. Rane, A.; Wilkinson, G.R.; Shand, D.G. Prediction of hepatic extraction ratio from in vitro measurement of intrinsic clearance. *J. Pharmacol. Exp. Ther*. **1977**, *200(2),* 420-424.

6. Davidsen, A.B.; Mardal, M.; Linnet, K. In vitro metabolism and hepatic intrinsic clearance of the synthetic cannabinoid receptor agonist JWH-122 and its four ω-halogenated analogues. *AAPS J.* **2019**, *21*, 1-9. doi: 10.1208/s12248-019-0338-6.

7. Iwatsubo, T.; Hirota, N.; Ooie, T.; Suzuki, H.; Shimada, N.; Chiba, K.; Ishizaki, T.; Green, C.E.; Tyson, C.A.; Sugiyama, Y. Prediction of in vivo drug metabolism in the human liver from in vitro metabolism data. *Pharmacol. Ther.* **1997**, *73,* 147-171. doi: 10.1016/s0163-7258(96)00184-2.

8. Wilson, Z.E.; Rostami-Hodjegan, A.; Burn, J.L.; Tooley, A.; Boyle, J.; Ellis, S.W.; Tucker, G.T. Inter-individual variability in levels of human microsomal protein and hepatocellularity per gram of liver. *Br. J. Clin. Pharmacol.* **2003,** 56, 433-440. doi: 10.1046/j.1365-2125.2003.01881.x.

9. Huang, Z.; Li, H.; Zhang, Q.; Tan, X.; Lu, F.; Liu, H., Li, S. Characterization of preclinical in vitro and in vivo pharmacokinetics properties for KBP-7018, a new tyrosine kinase inhibitor candidate for treatment of idiopathic pulmonary fibrosis. *Drug Des. Devel. Ther.* **2015,** *9,* 4319-4328. doi: 10.2147/DDDT.S83055.

Section S5

Plasma stability study data

The human plasma used was pooled from 50 donors, and was collected as whole blood from donors in the United States at an FDA-approved collection center, processed into plasma by centrifugation and immediately frozen. Plasma was tested by the manufacturers for a range of FDA-required viral markers.

The stability of the tested SCRAs, select metabolites and procaine control in pooled human plasma over 3-5 hours under physiological conditions (37 ℃, pH 7.4), both in the presence and absence of esterase inhibitors bis(4-nitrophenyl) phosphate (BNPP) and phenyl methyl sulphonyl fluoride (PMSF) is detailed in figures S2-S20 below (n≥3 ± SD).

Figure S2 procaine plasma stability (control), 5 hours, n=3, error bars represent ± standard deviation

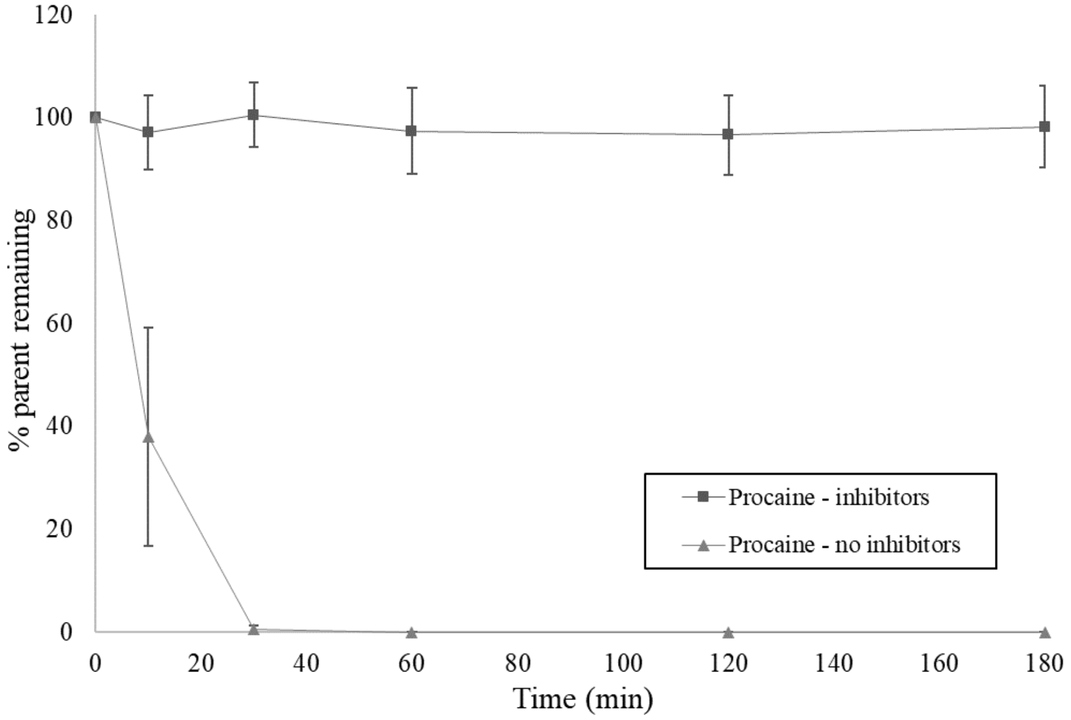

<u>Figure S3</u> <u>(*S*)-AMB-FUBINACA (**1**) plasma stability, 5 hours, n=3 (with inhibitors); n=4 (no inhibitors), error bars represent ± standard deviation</u>

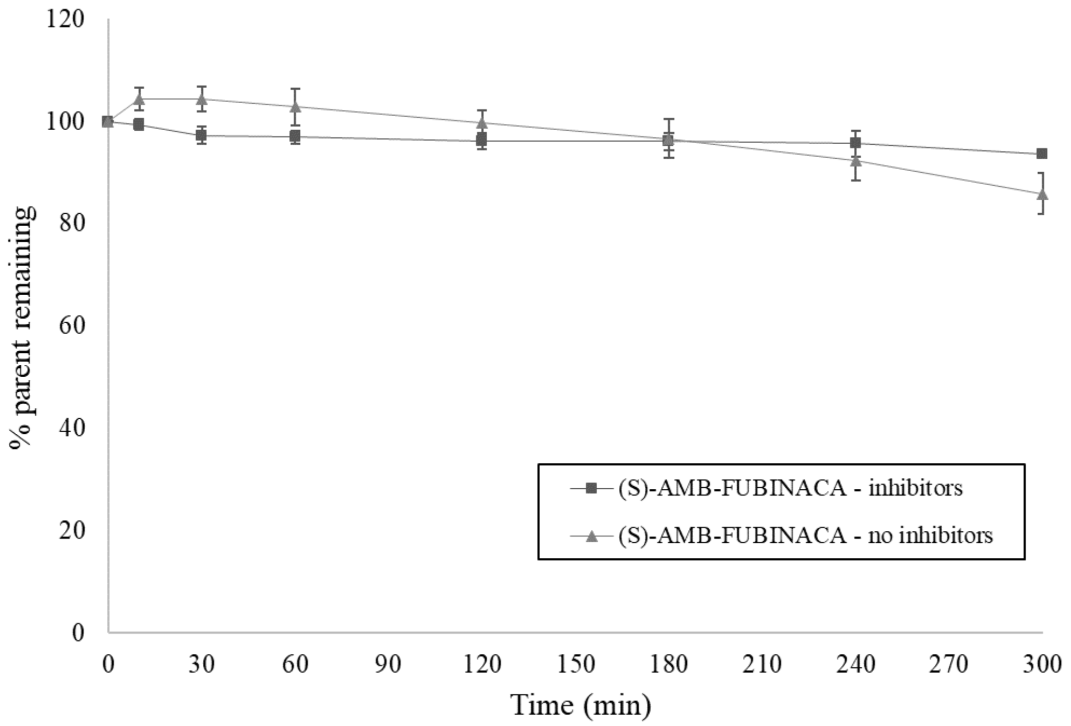

<u>Figure S4</u> <u>(*S*)-5F-MDMB-PINACA plasma stability, 3 hours, n=3 (with inhibitors); n=7 (no inhibitors), error bars represent ± standard deviation</u>

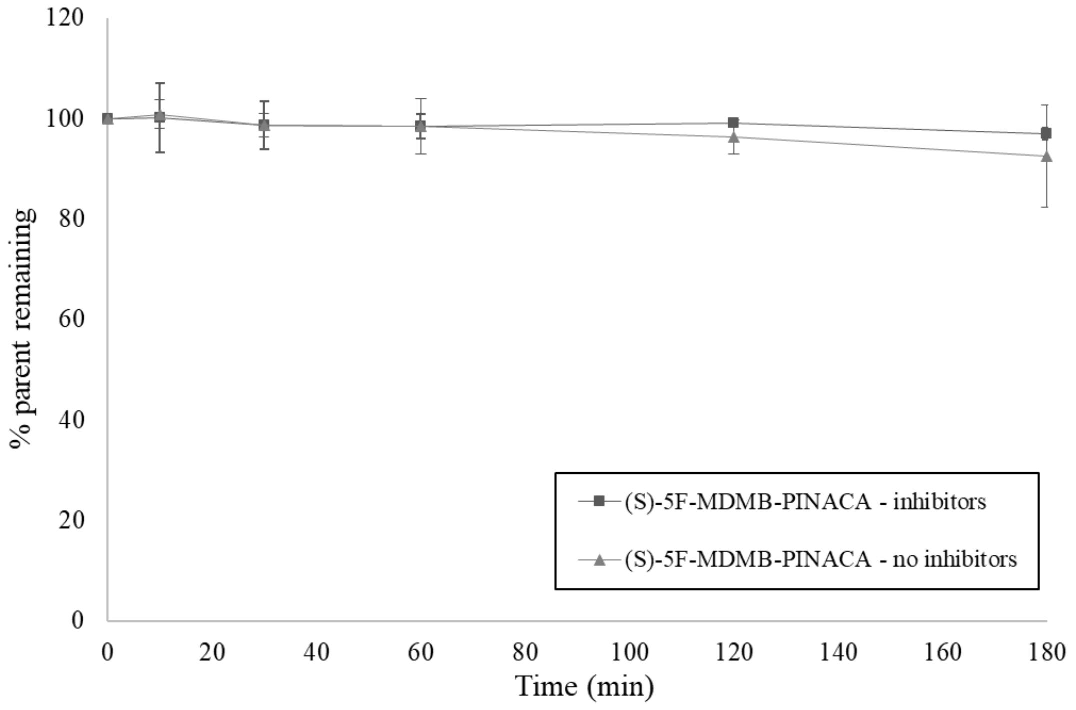

Figure S5 (*R*)-5F-MDMB-PINACA plasma stability, 3 hours, n=7 (with inhibitors); n=6 (no inhibitors), error bars represent ± standard deviation

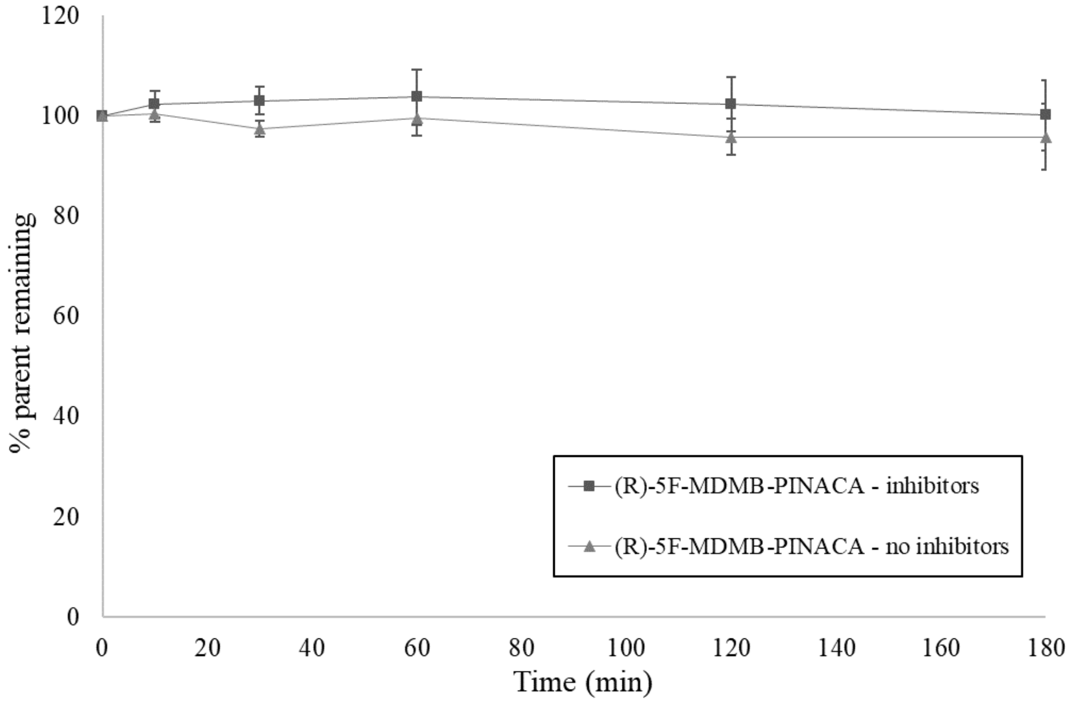

Figure S6 (*S*)-5F-MDMB-PICA plasma stability, 3 hours, n=3, error bars represent ± standard deviation

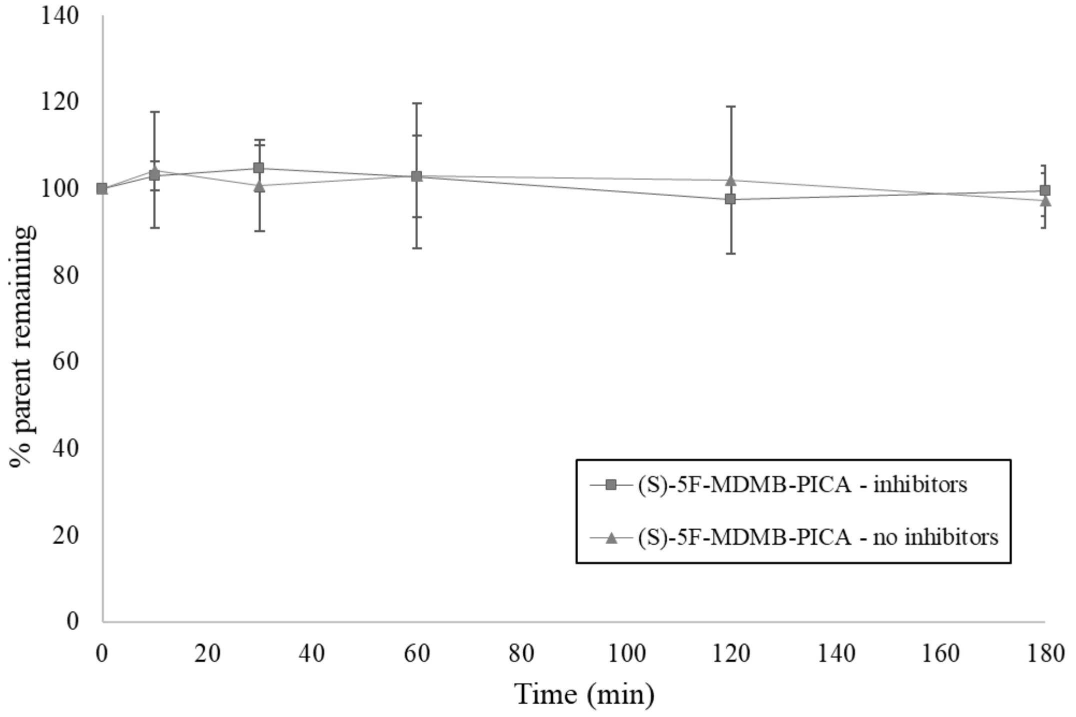

Figure S7 (*R*)-5F-MDMB-PICA plasma stability, 3 hours, n=3, error bars represent ± standard deviation

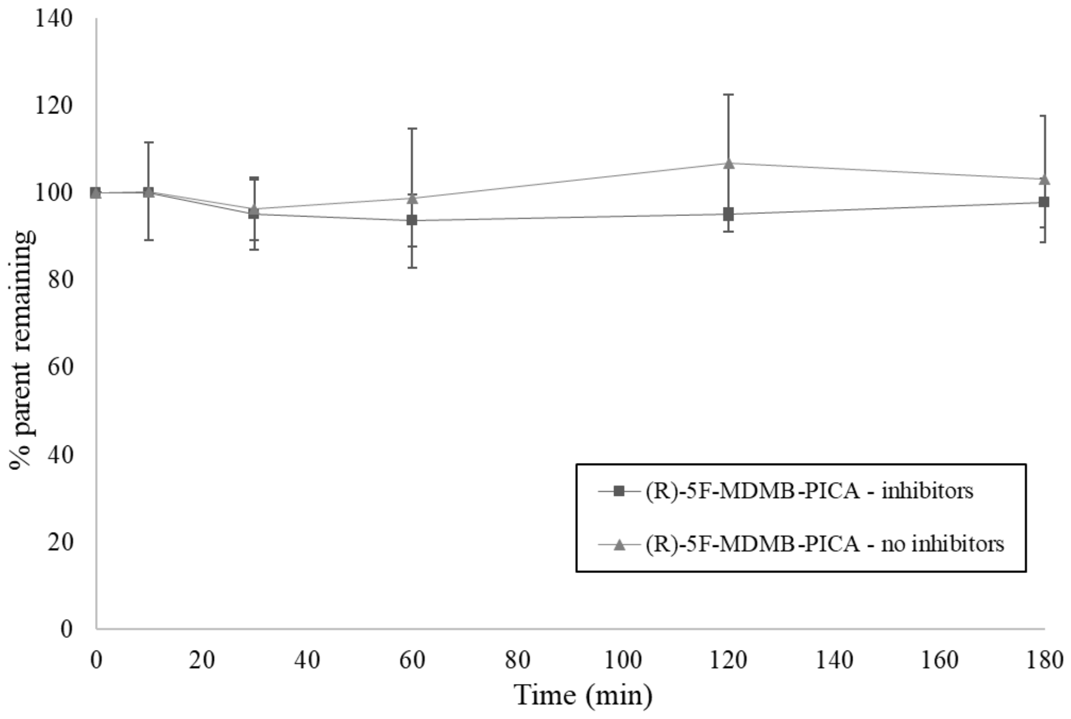

Figure S8 (*S*)-4F-MDMB-BINACA plasma stability, 3 hours, n=3, error bars represent ± standard deviation

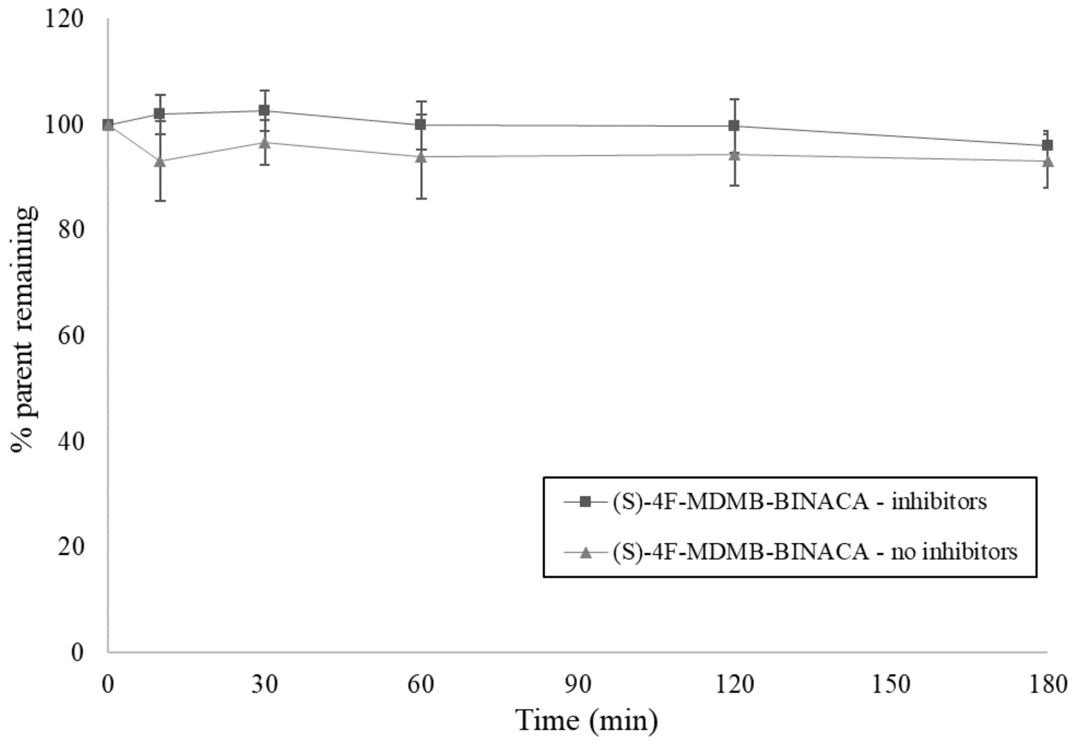

Figure S9 (*R*)-4F-MDMB-BINACA plasma stability, 3 hours, n=3, error bars represent ± standard deviation

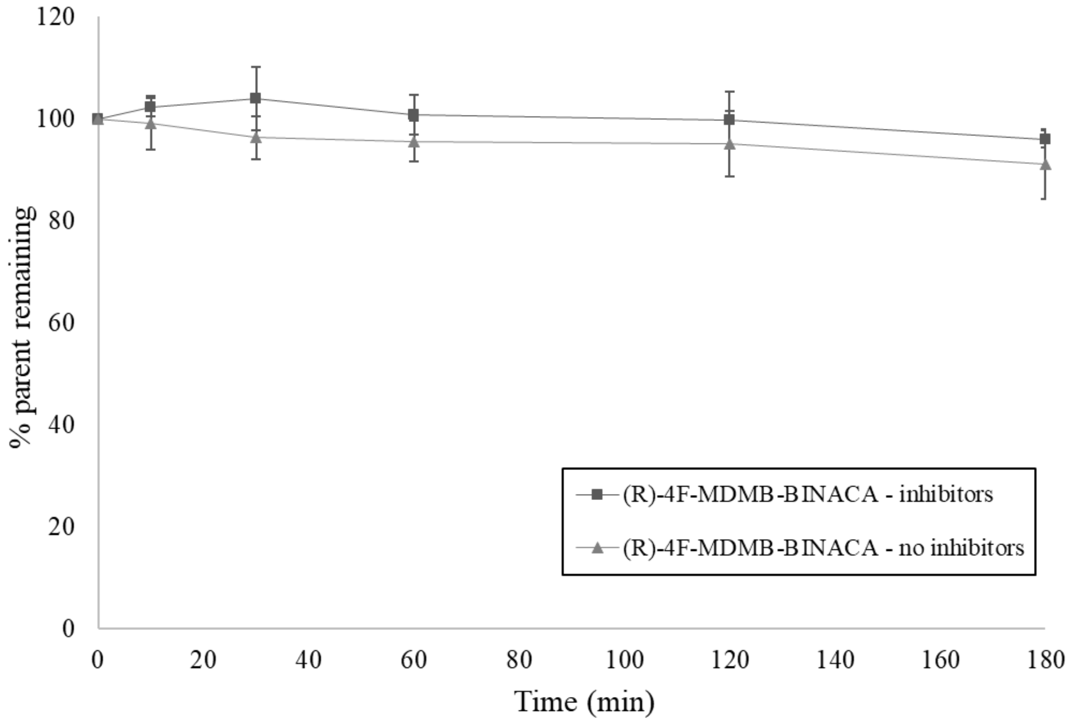

Figure S10 (*S*)-MDMB-4en-PINACA plasma stability, 3 hours, n=3, error bars represent ± standard deviation

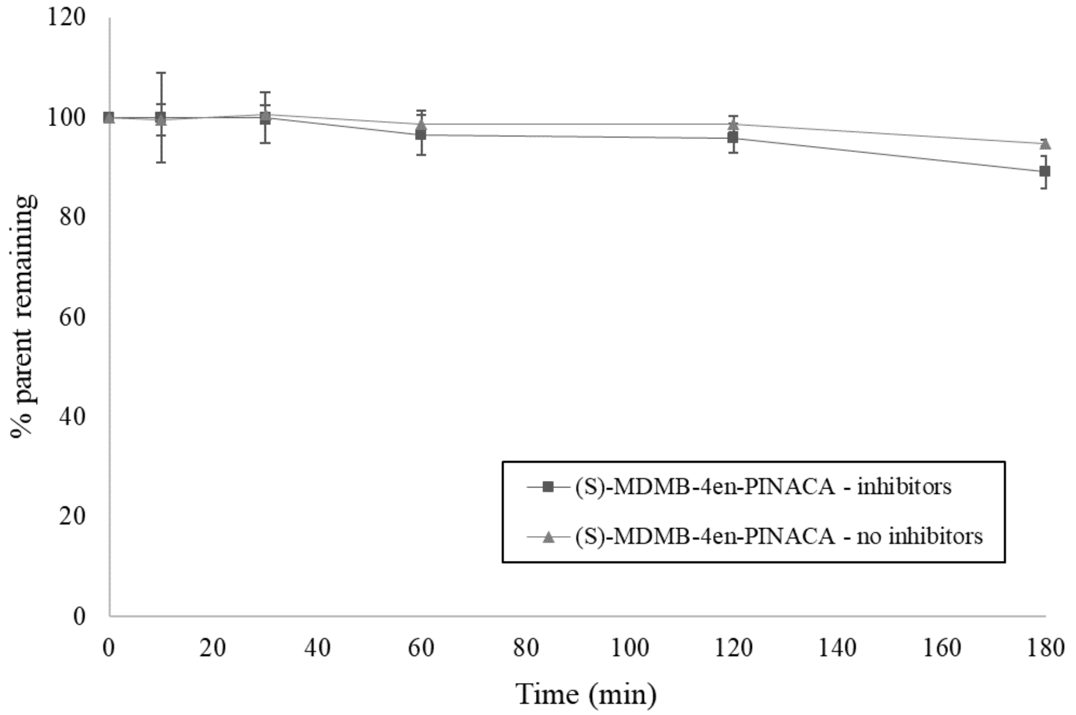

Figure S11 (*R*)-MDMB-4en-PINACA plasma stability, 3 hours, n=3, error bars represent ± standard deviation

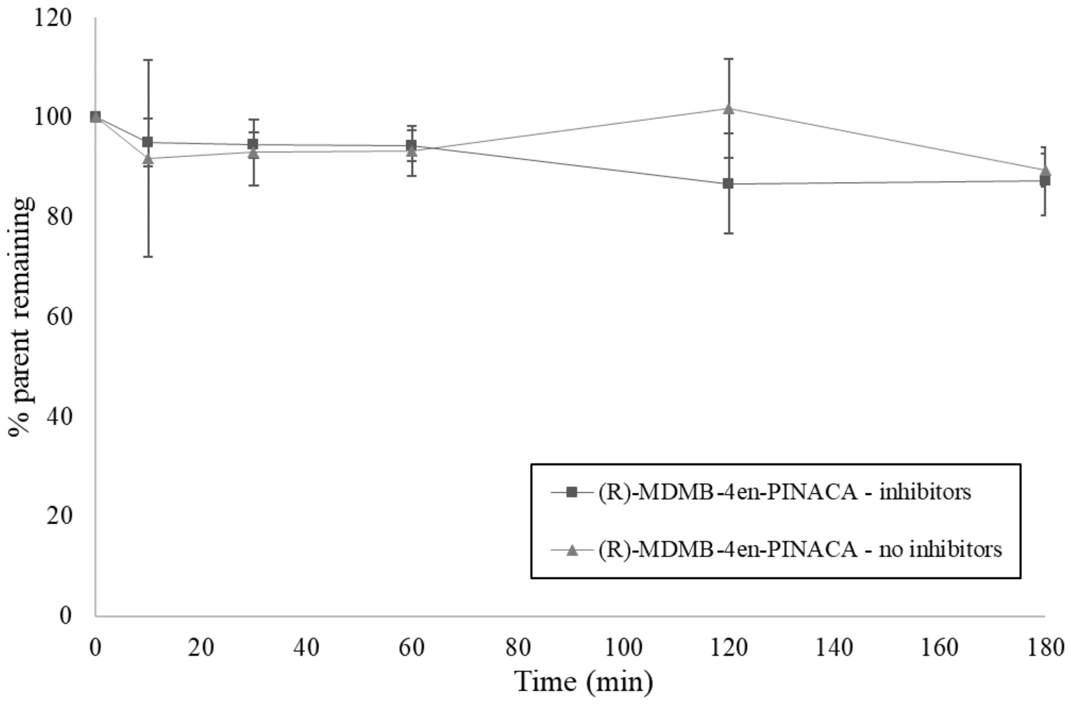

Figure S12 (*S*)-MDMB-FUBINACA plasma stability, 3 hours, n=3, error bars represent ± standard deviation

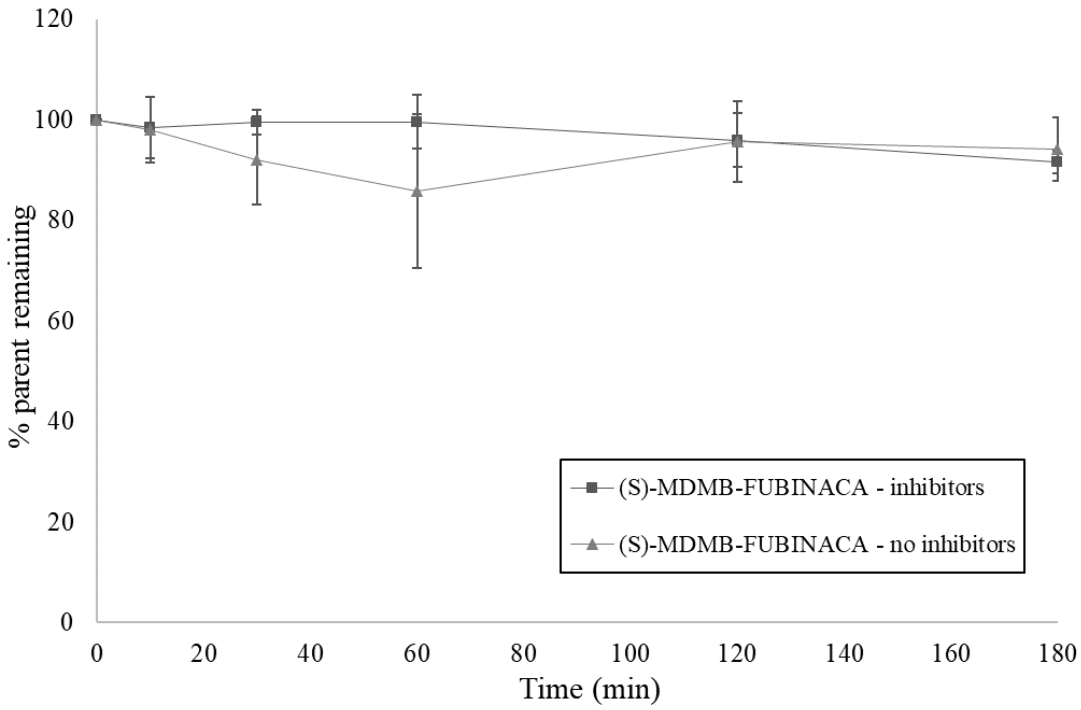

Figure S13 (*S*)-AMB-4en-PICA plasma stability, 3 hours, n=3, error bars represent ±standard deviation

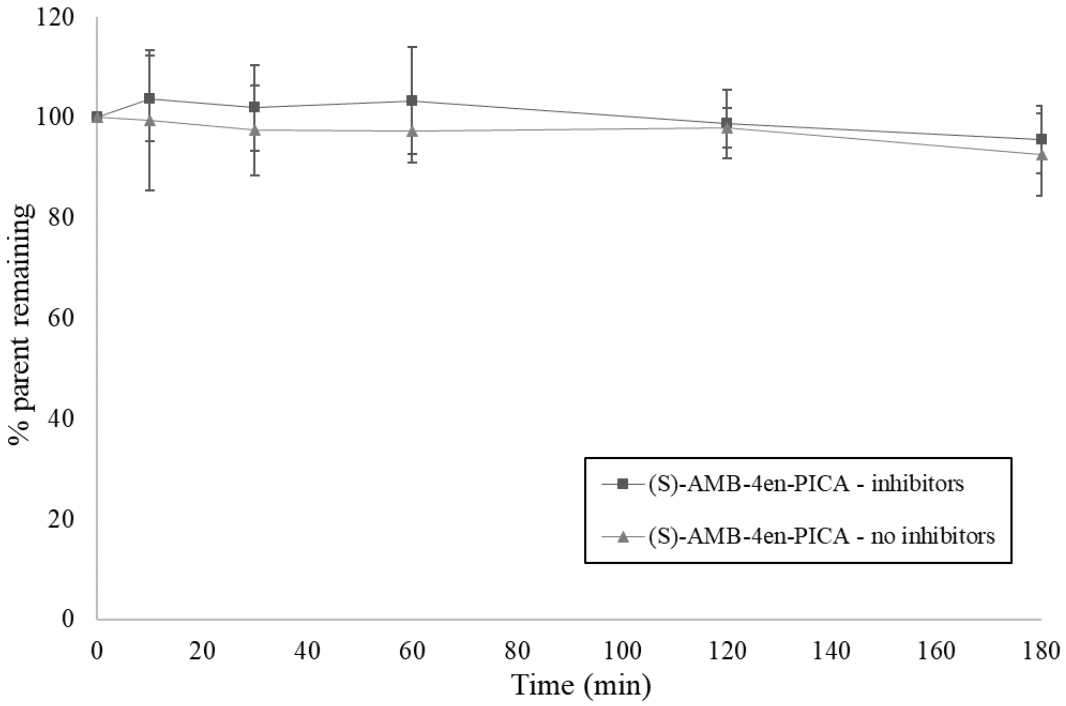

Figure S14 (*R*)-AMB-4en-PICA plasma stability, 3 hours, n=3, error bars represent ±standard deviation

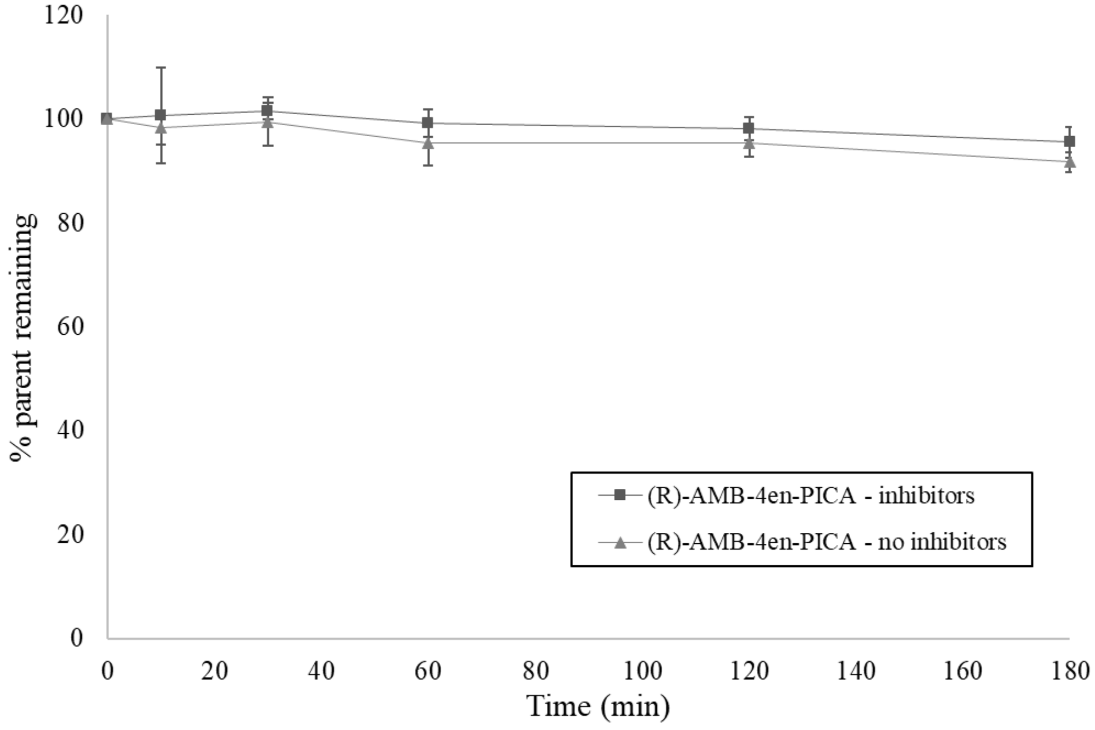

Figure S15 (*S*)-AB-FUBINACA plasma stability, 3 hours, n=3, error bars represent ± standard deviation

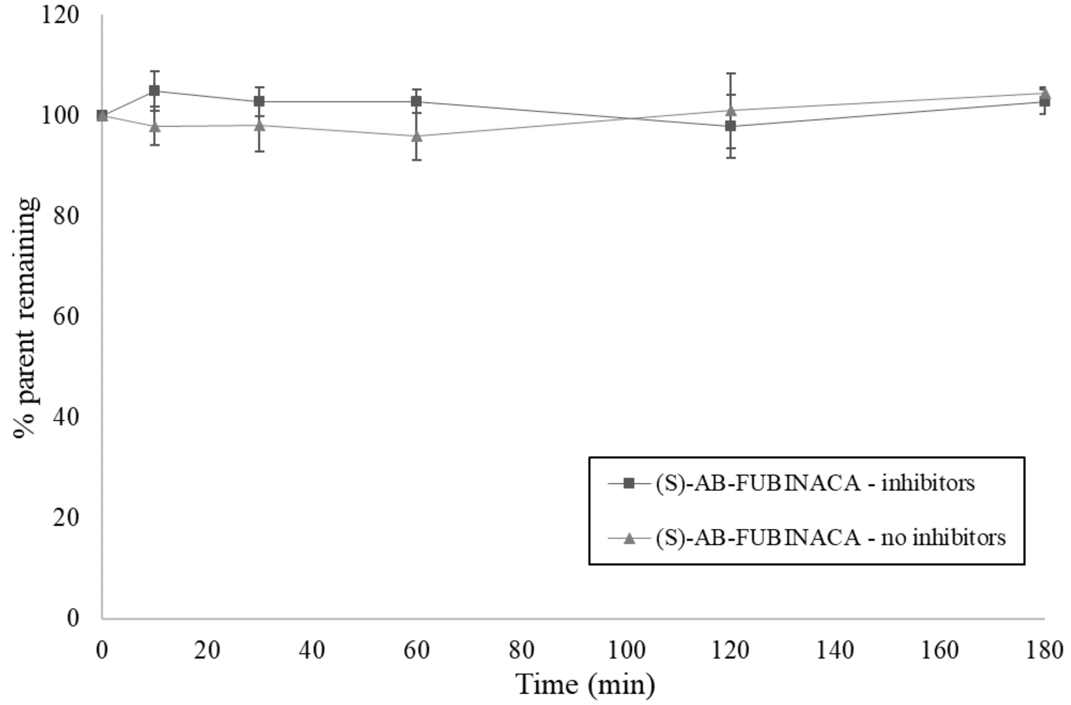

Figure S16 (*S*)-AB-CHMINACA plasma stability, 3 hours, n=3, error bars represent ± standard deviation

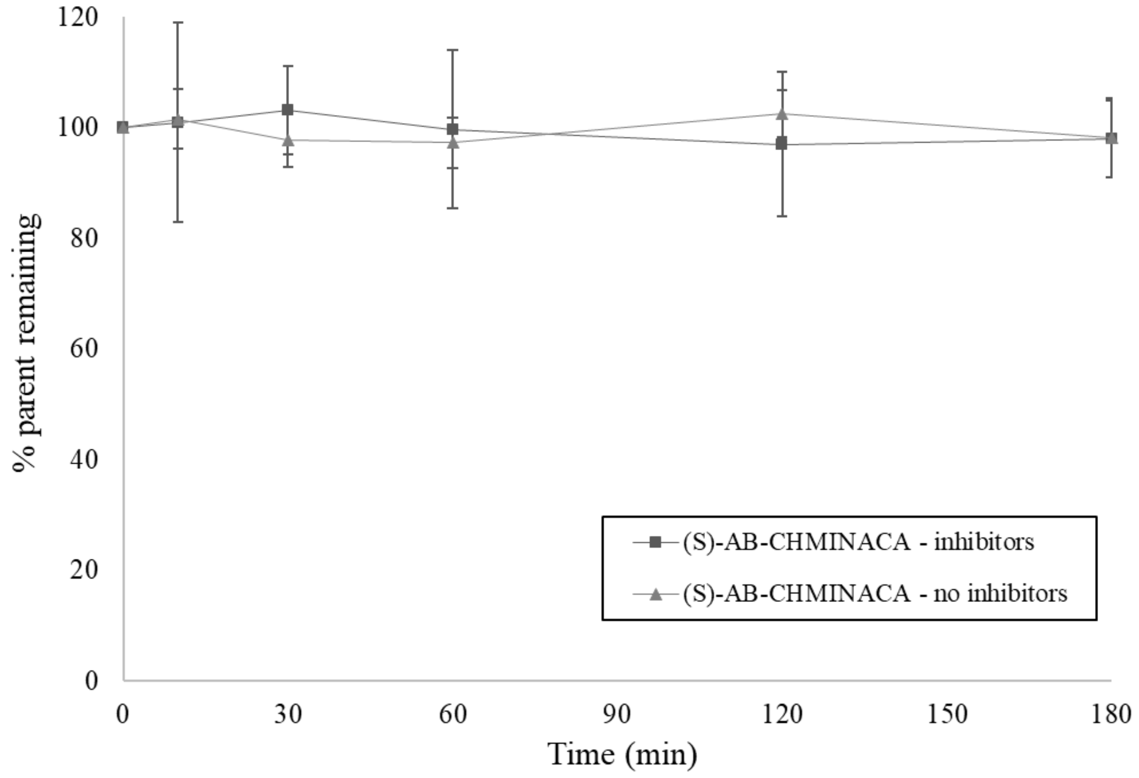

Figure S17 (*S*)-AMB-CHMICA plasma stability, 3 hours, n=3, error bars represent ±standard deviation

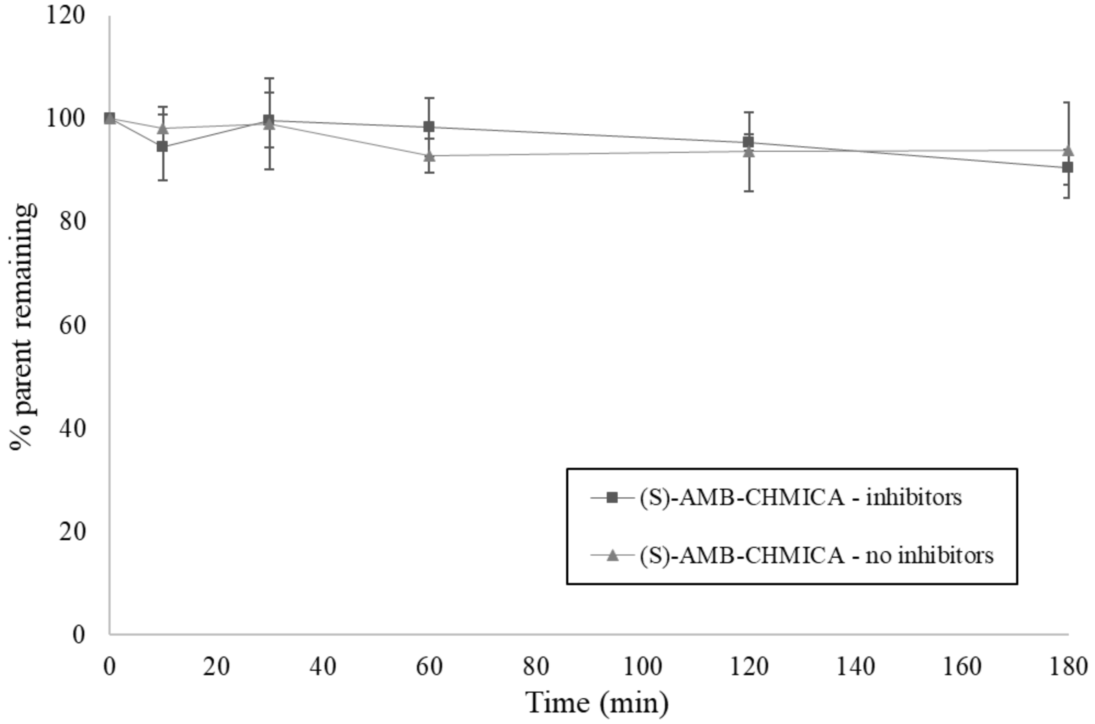

Figure S18 (*S*)-5F-AMB-PINACA plasma stability, 3 hours, n=3, error bars represent ±standard deviation

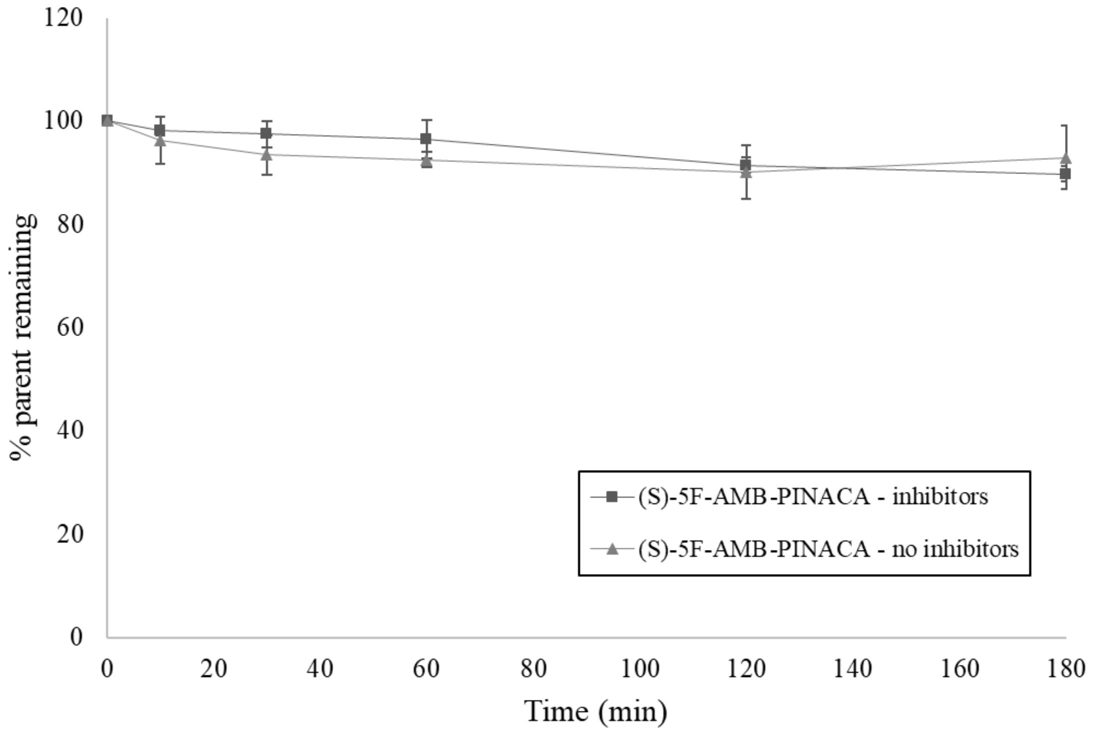

Figure S19 (*S*)-MDMB-4en-PICA plasma stability, 3 hours, n=3, error bars represent ± standard deviation

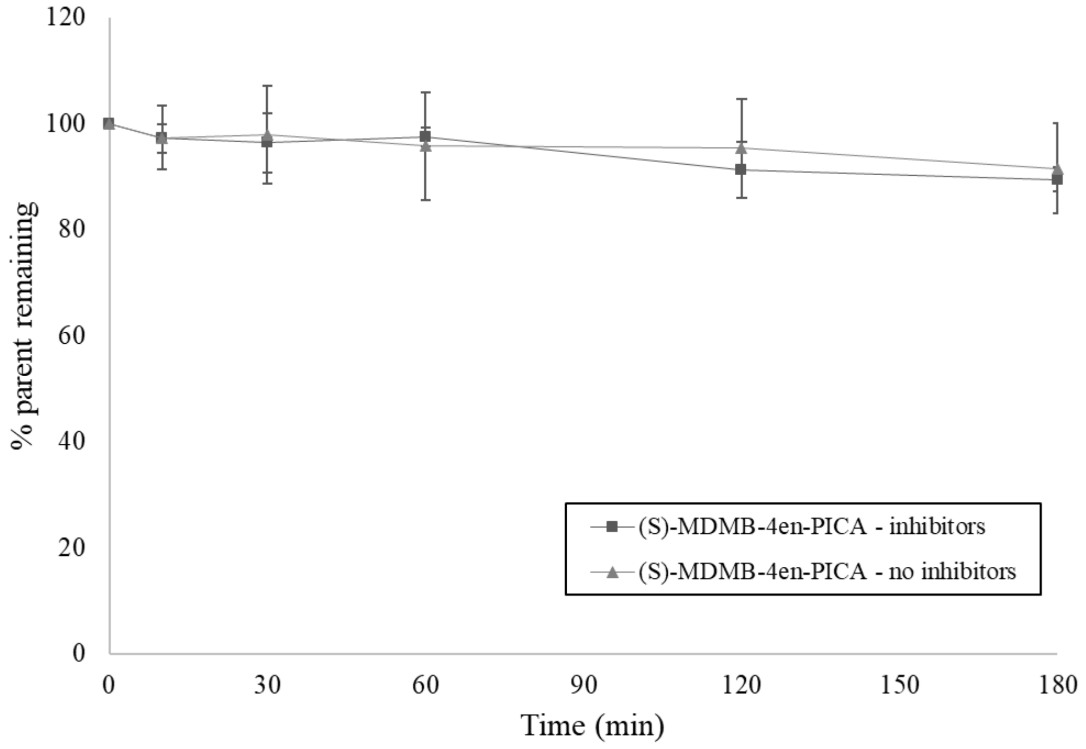

Figure S20 (*R*)-MDMB-4en-PICA plasma stability, 3 hours, n=3, error bars represent ± standard deviation

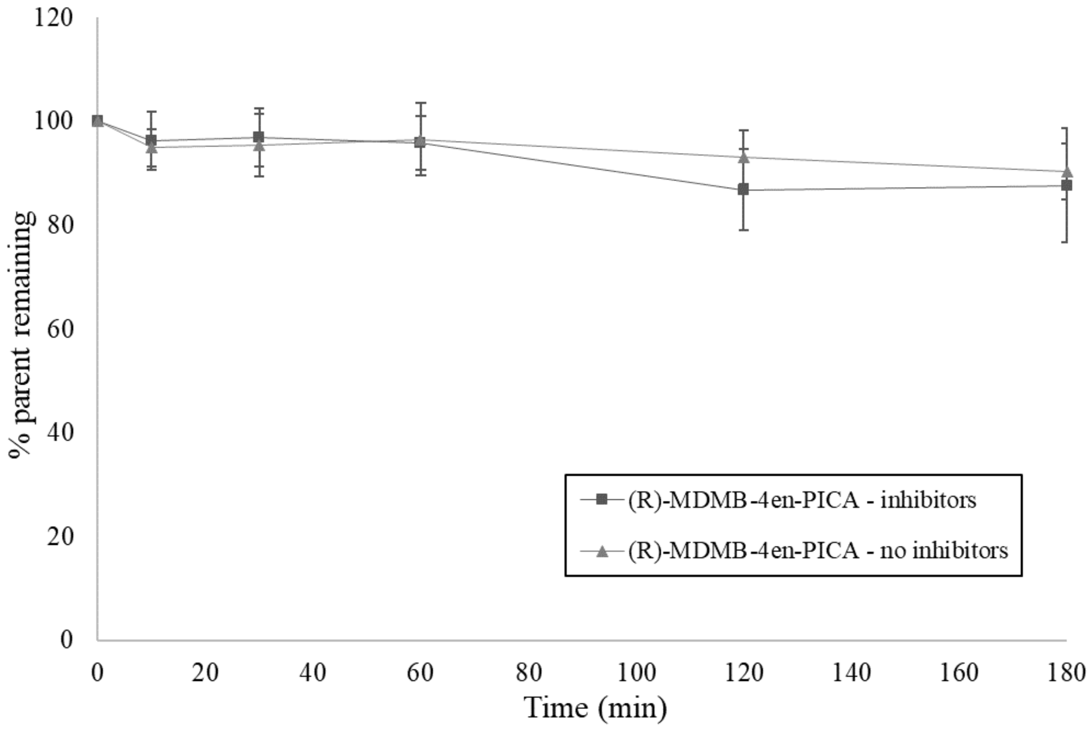

Section S6

Intrinsic clearance data – pooled human liver microsome incubations

The percentage of parent compound remaining, based on peak area ratio of analyte/internal standard (donepezil) in pooled human liver microsome (pHLM) incubations is detailed below in figures S21-33, with enantiomers of each compound overlaid for comparison. Data for each replicate is provided in table S4-28. Intrinsic clearance and predicted *in vivo* data calculated using alternative microsomal scaling factors found in the literature – 32 [10] and 45 [11] mg microsomal protein per gram liver – are provided in tables S29 and S30, respectively.

Figure S21 Verapamil (control) clearance (HLM), 30 minutes, n=14, error bars represent ± standard deviation

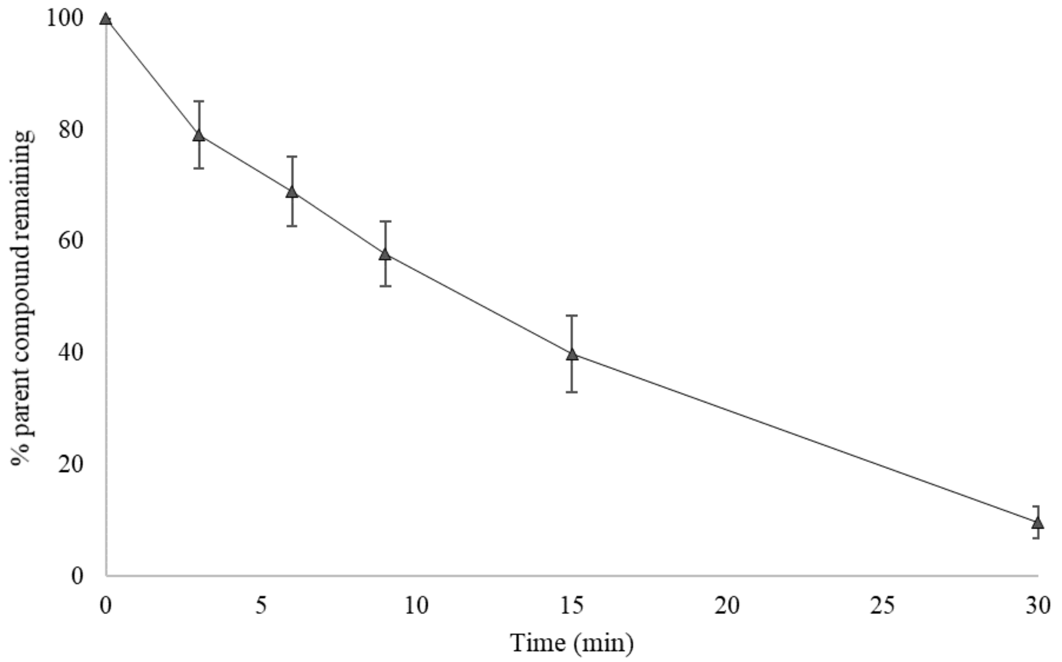

<u>**Figure S22** AMB-FUBINACA clearance (HLM), 30 minutes, n=3 ((*S*)-); n=6 ((*R*)-), error bars represent ± standard deviation</u>

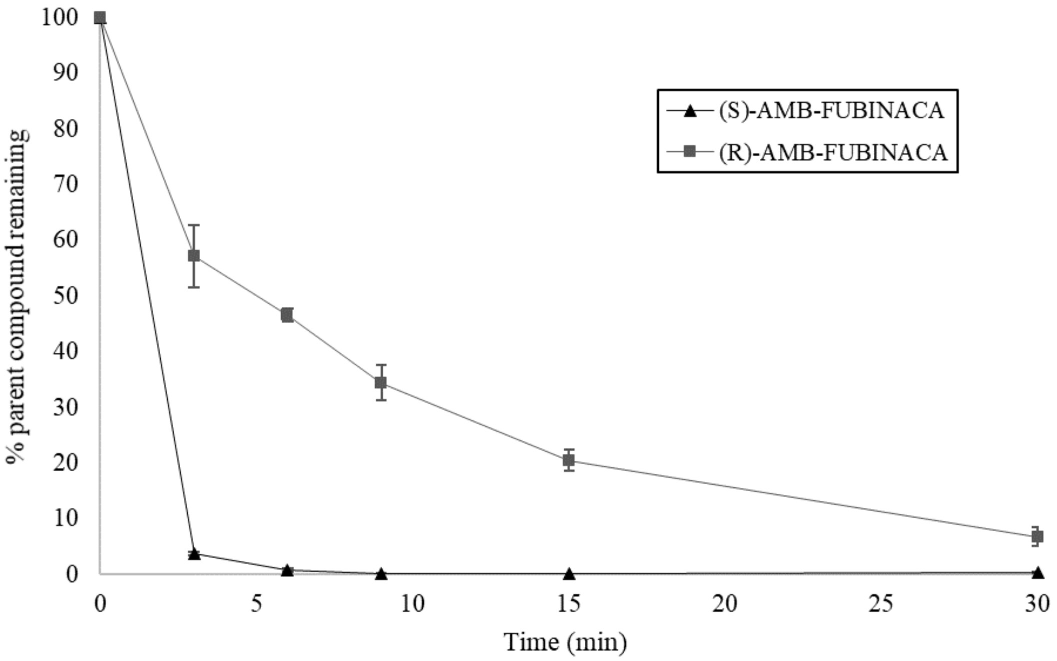

<u>**Figure S23** 5F-MDMB-PINACA clearance (HLM), 30 minutes, n=5, error bars represent ± standard deviation</u>

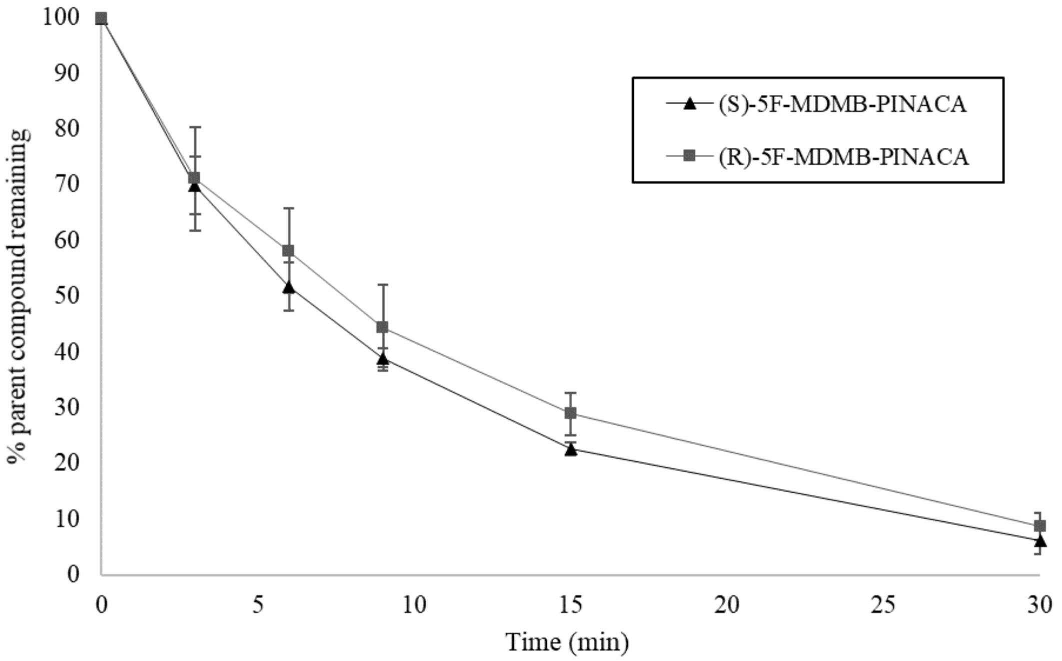

Figure S24 5F-MDMB-PICA clearance (HLM), 30 minutes, n=3, error bars represent ± standard deviation

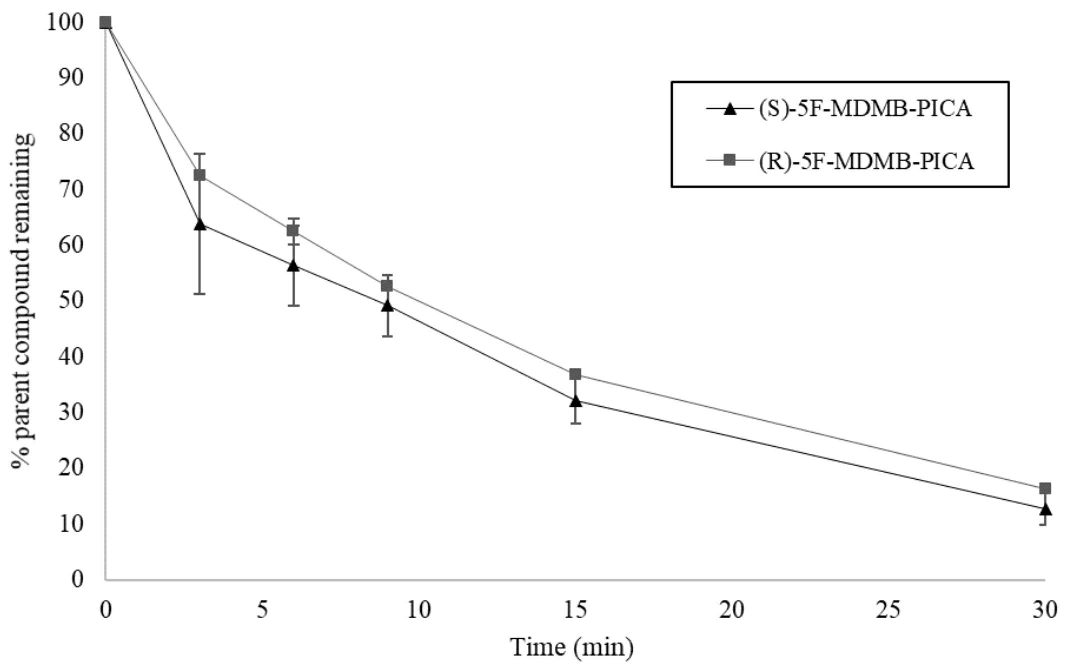

Figure S25 4F-MDMB-BINACA clearance (HLM), 30 minutes, n=3, error bars represent ± standard deviation

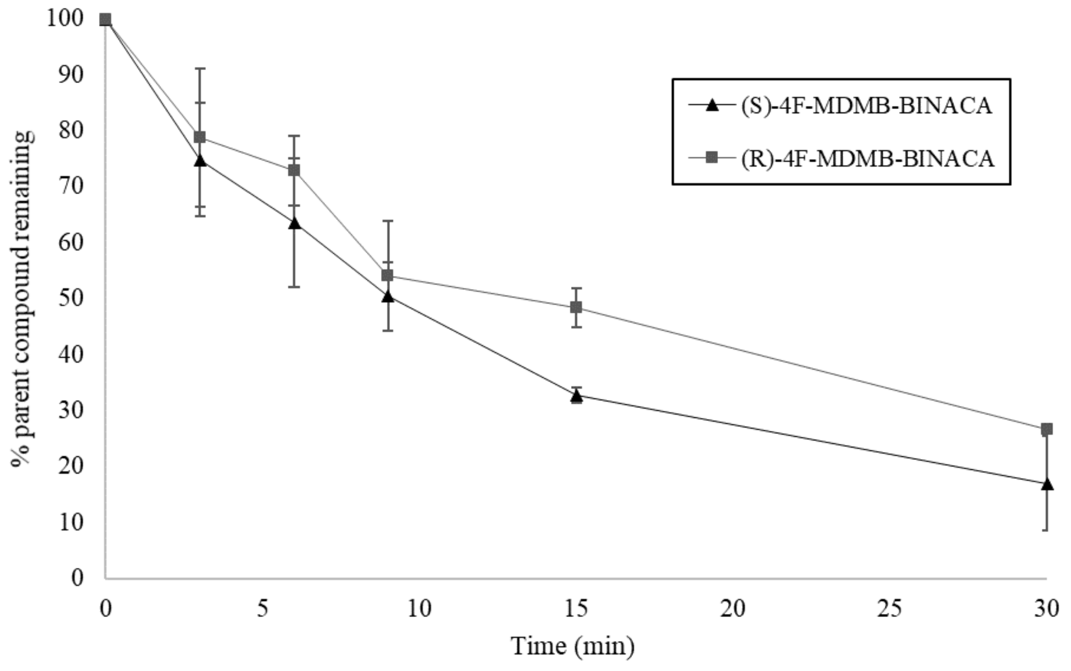

Figure S26 MDMB-4en-PINACA clearance (HLM), 30 minutes, n=7, error bars represent ±standard deviation

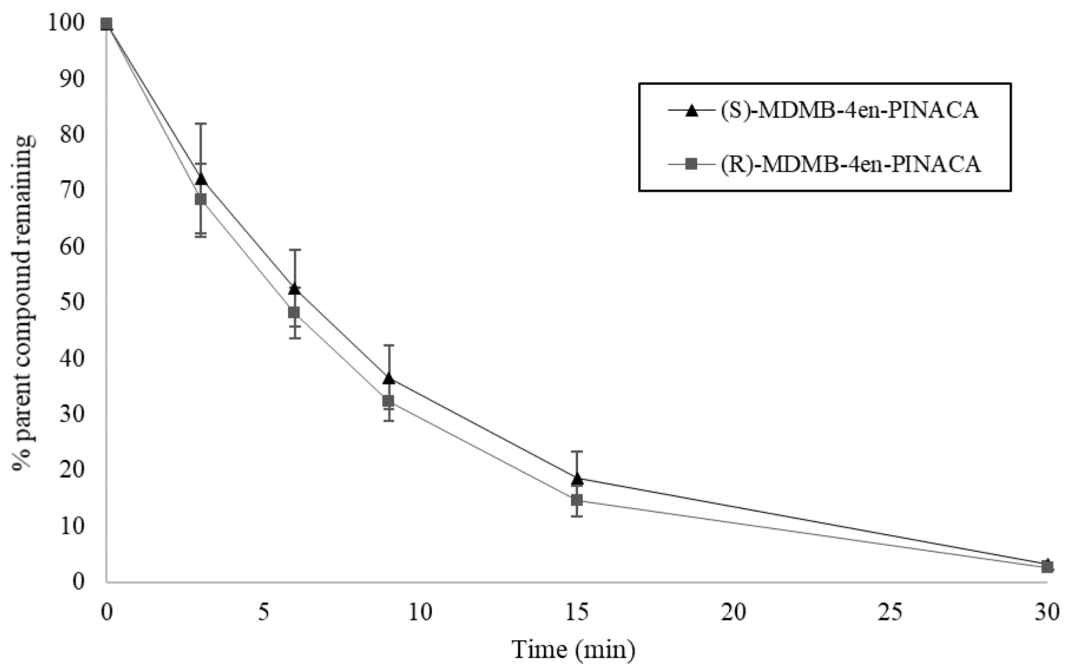

Figure S27 AB-FUBINACA clearance (HLM), 30 minutes, n=3, error bars represent ±standard deviation

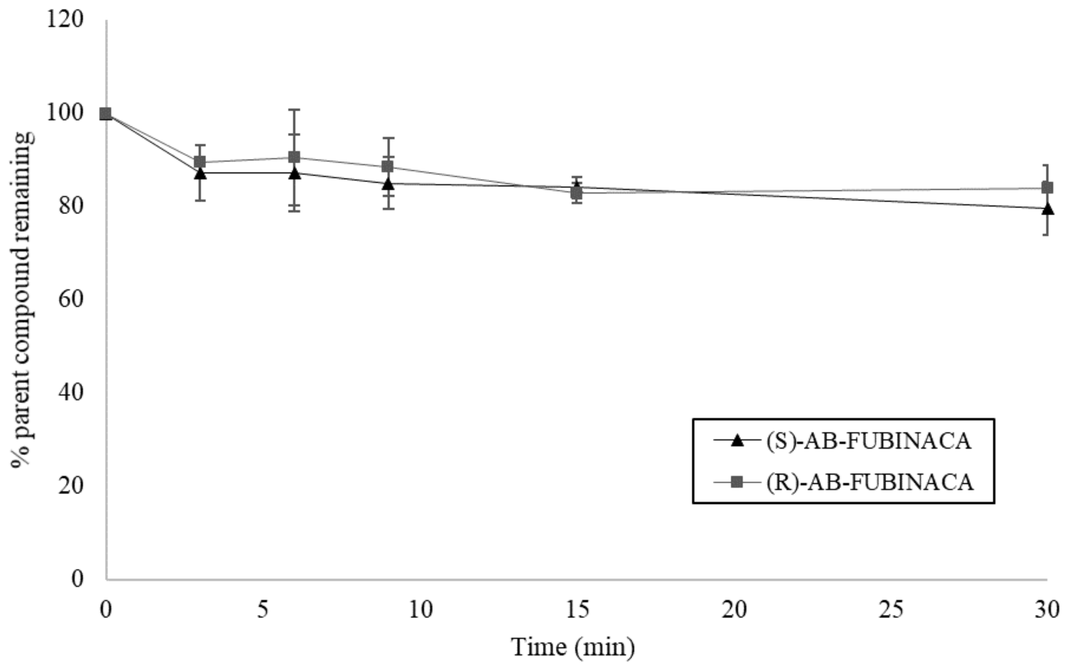

Figure S28 AMB-4en-PICA clearance (HLM), 30 minutes, n=10 ((*S*)-); n=11 ((*R*)-), error bars represent ± standard deviation

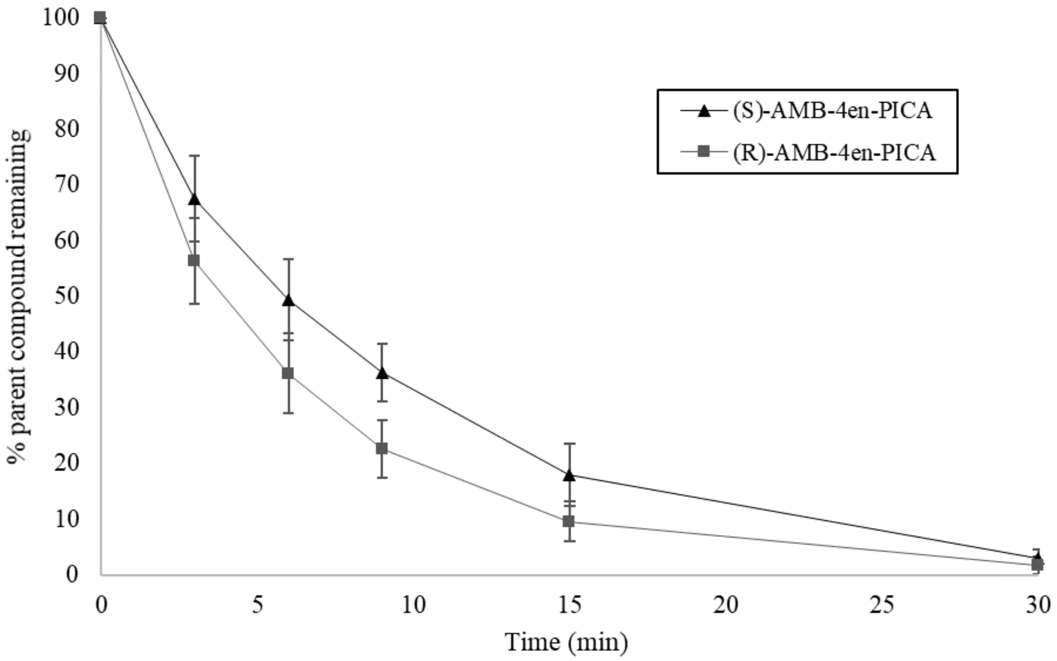

Figure S29 MDMB-FUBINACA clearance (HLM), 30 minutes, n=6, error bars represent ± standard deviation

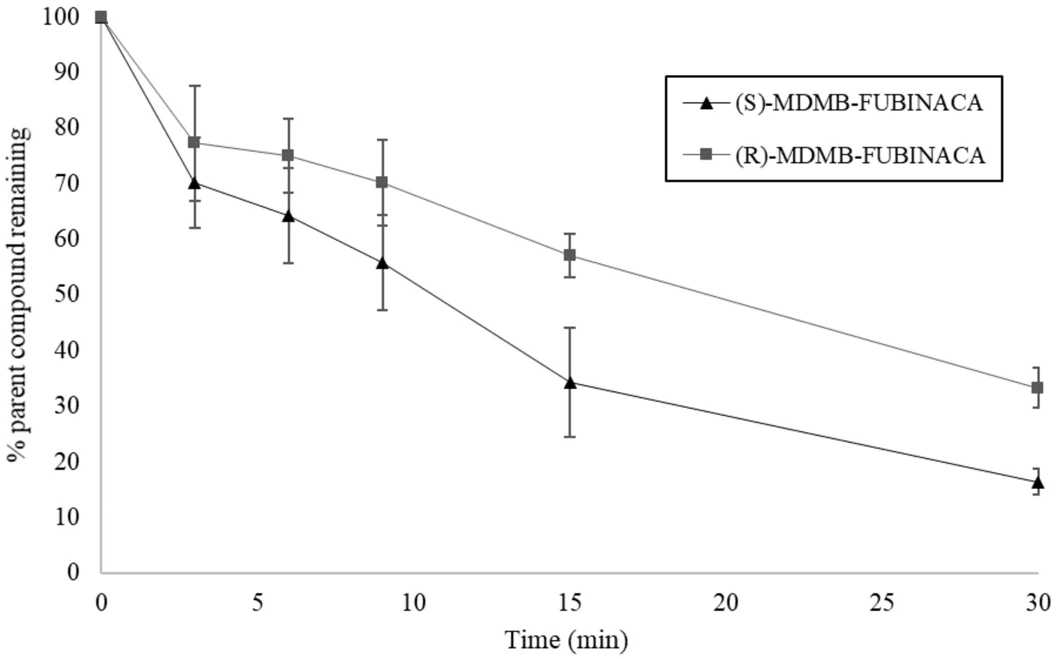

Figure S30 AB-CHMINACA clearance (HLM), 30 minutes, n=3, error bars represent ± standard deviation

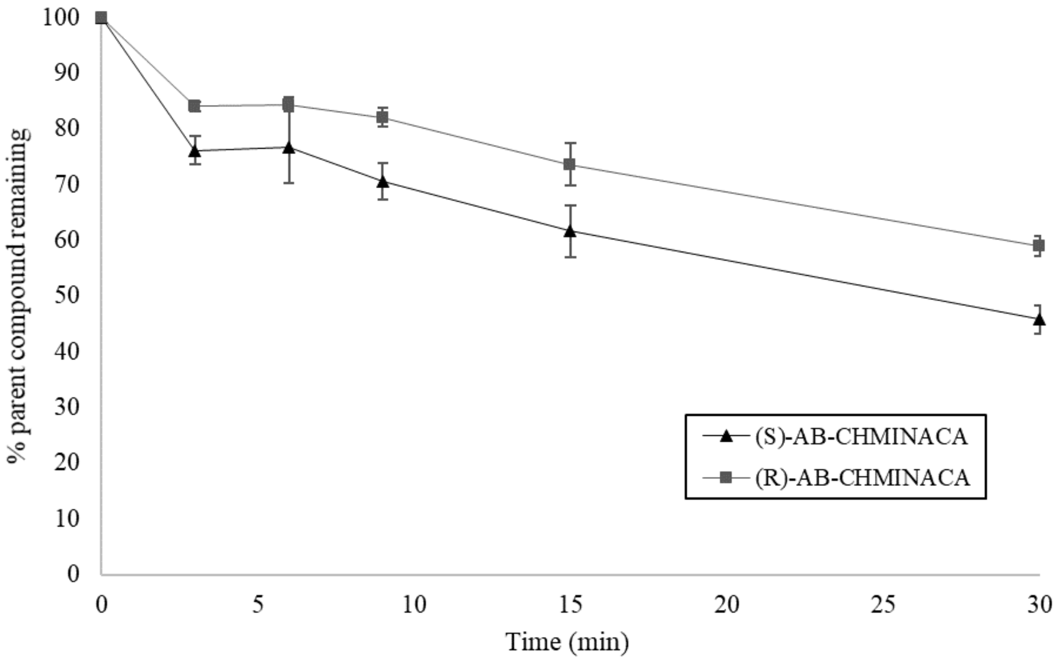

Figure S31 AMB-CHMICA clearance (HLM), 30 minutes, n=3, error bars represent ± standard deviation

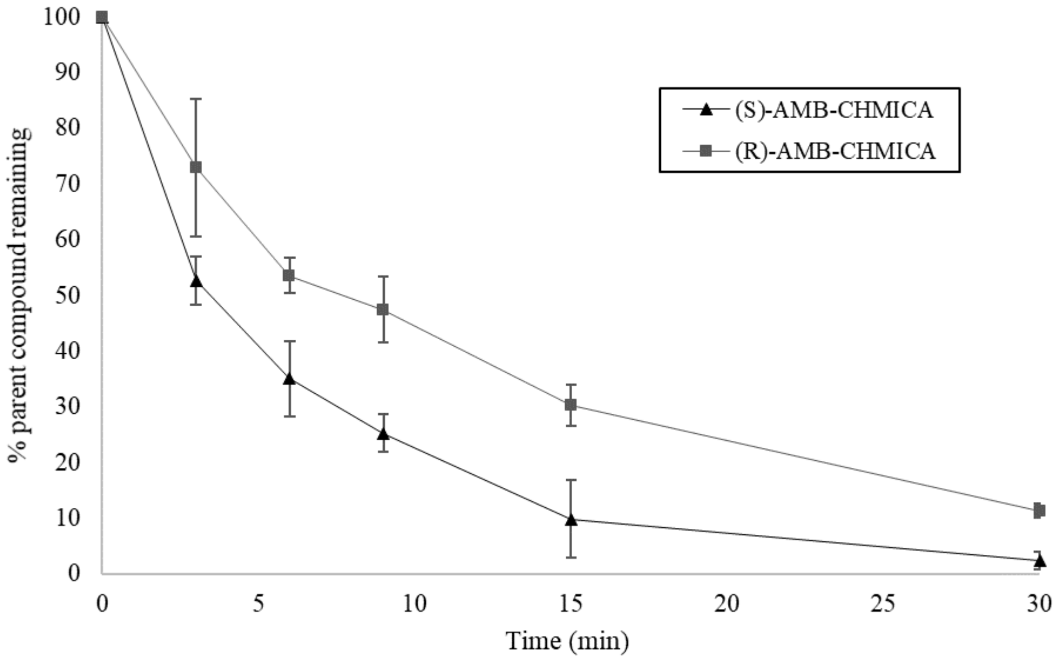

Figure S32 5F-AMB-PINACA clearance (HLM), 30 minutes, n=6, error bars represent ± standard deviation

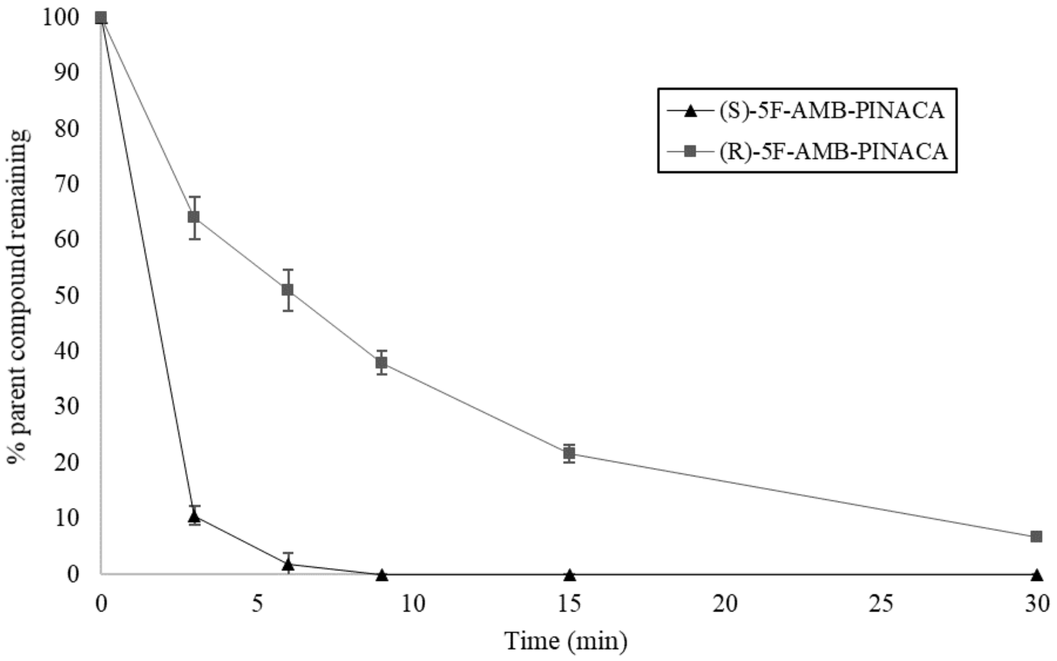

Figure S33 MDMB-4en-PICA clearance (HLM), 30 minutes, n=11 ((*S*)-); n=9 ((*R*)-), error bars represent ± standard deviation

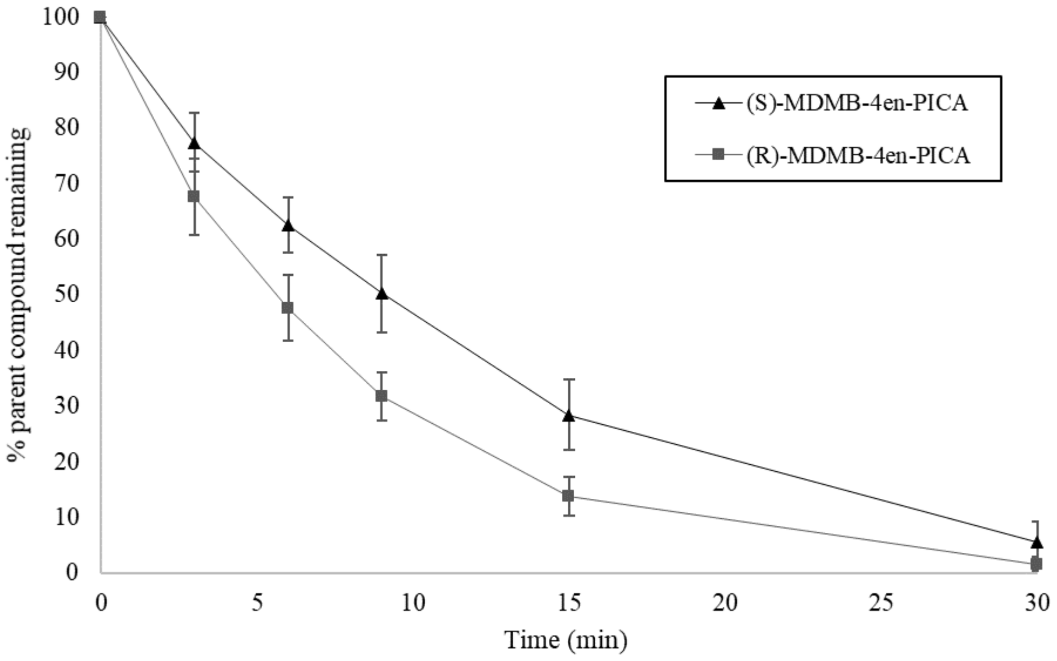

Table S4 Verapamil (control) percentage remaining (pHLM)

Time (min)	% parent compound remaining															
	1	2	3	4	5	6	7	8	9	10	11	12	13	14	Average	SD
0	100.0	100.0	100.0	100.0	100.0	100.0	100.0	100.0	100.0	100.0	100.0	100.0	100.0	100.0	100.0	-
3	74.7	86.9	74.5	78.2	80.2	77.5	70.9	72.8	85.1	84.5	87.3	87.2	73.9	73.8	79.1	6.0
6	63.5	72.6	67.6	68.9	71.0	63.4	62.9	59.3	75.6	77.8	78.6	74.5	64.9	64.5	68.9	6.2
9	57.7	62.6	57.2	58.8	60.4	52.2	49.8	49.1	64.2	61.5	67.3	63.4	53.9	51.5	57.8	5.8
15	39.7	45.8	43.2	42.5	41.7	31.4	30.6	31.1	47.9	46.2	48.2	44.4	33.4	31.1	39.8	6.8
30	11.0	13.6	12.4	11.1	11.4	5.4	5.8	6.0	11.4	11.8	11.1	10.6	7.7	6.4	9.7	2.8

Table S5 (*S*)-AMB-FUBINACA percentage remaining (pHLM)

Time (min)	% parent compound remaining				
	1	2	3	Average	SD
0	100.0	100.0	100.0	100.0	-
3	4.1	3.3	3.8	3.8	0.4
6	0.8	1.0	0.4	0.7	0.3
9	0.0	0.2	0.4	0.2	0.2
15	0.1	0.1	0.1	0.1	0.0
30	0.2	0.4	0.3	0.3	0.1

Table S6 (*R*)-AMB-FUBINACA percentage remaining (pHLM)

Time (min)	% parent compound remaining							
	1	2	3	4	5	6	Average	SD
0	100.0	100.0	100.0	100.0	100.0	100.0	100.0	-
3	56.1	46.8	59.2	61.9	62.2	56.7	57.2	5.7
6	46.8	44.5	47.2	46.9	46.9	47.8	46.7	1.1
9	32.1	31.3	31.6	38.1	37.8	35.8	34.5	3.2
15	17.8	18.9	21.1	23.4	20.9	21.0	20.5	1.9
30	5.2	4.9	5.9	8.6	8.1	8.0	6.8	1.7

Table S7 (*S*)-5F-MDMB-PINACA percentage remaining (pHLM)

Time (min)	% parent compound remaining						
	1	2	3	4	5	Average	SD
0	100.0	100.0	100.0	100.0	100.0	100.0	-
3	69.4	68.1	62.5	76.0	73.6	69.9	5.2
6	50.0	49.1	47.2	57.8	54.4	51.7	4.3
9	36.7	38.0	38.6	41.0	40.4	39.0	1.7
15	23.8	22.8	20.9	23.7	22.3	22.7	1.2
30	7.3	7.9	8.2	4.0	3.1	6.1	2.4

Table S8 (*R*)-5F-MDMB-PINACA percentage remaining (pHLM)

Time (min)	% parent compound remaining						
	1	2	3	4	5	Average	SD
0	100.0	100.0	100.0	100.0	100.0	100.0	-
3	64.7	65.1	63.7	78.2	84.0	71.1	9.3
6	50.8	51.8	56.1	64.0	68.1	58.2	7.6
9	39.0	36.9	41.2	50.4	54.7	44.4	7.7
15	25.5	26.6	26.3	31.9	34.1	28.9	3.8
30	10.1	10.4	10.9	6.5	5.7	8.7	2.4

Table S9 (*S*)-5F-MDMB-PICA percentage remaining (pHLM)

Time (min)	% parent compound remaining				
	1	2	3	Average	SD
0	100.0	100.0	100.0	100.0	-
3	70.3	49.5	71.8	63.9	12.5
6	60.1	48.0	60.9	56.3	7.2
9	49.6	43.4	54.5	49.2	5.6
15	35.6	27.6	33.2	32.1	4.1
30	14.9	9.6	13.7	12.8	2.8

Table S10 (*R*)-5F-MDMB-PICA percentage remaining (pHLM)

Time (min)	% parent compound remaining				
	1	2	3	Average	SD
0	100.0	100.0	100.0	100.0	-
3	72.7	73.0	72.0	72.6	0.5
6	65.1	61.2	61.2	62.5	2.3
9	52.3	52.2	53.5	52.6	0.7
15	37.4	36.6	36.4	36.8	0.5
30	15.5	16.5	17.2	16.4	0.8

Table S11 (*S*)-4F-MDMB-BINACA percentage remaining (pHLM)

Time (min)	% parent compound remaining				
	1	2	3	Average	SD
0	100.0	100.0	100.0	100.0	-
3	69.6	68.5	86.5	74.9	10.1
6	57.1	56.8	76.9	63.6	11.5
9	48.7	45.3	57.2	50.4	6.1
15	31.7	32.2	34.3	32.7	1.4
30	11.7	12.7	26.7	17.0	8.4

Table S12 (*R*)-4F-MDMB-BINACA percentage remaining (pHLM)

Time (min)	% parent compound remaining				
	1	2	3	Average	SD
0	100.0	100.0	100.0	100.0	-
3	68.3	92.3	75.9	78.8	12.3
6	78.8	73.2	66.4	72.8	6.2
9	42.9	60.0	59.5	54.1	9.7
15	52.1	45.3	47.8	48.4	3.5
30	27.2	26.3	26.3	26.6	0.5

Table S13 (*S*)-MDMB-4en-PINACA percentage remaining (pHLM)

Time (min)	% parent compound remaining								
	1	2	3	4	5	6	7	Average	SD
0	100.0	100.0	100.0	100.0	100.0	100.0	100.0	100.0	-
3	59.8	62.6	64.4	77.8	81.2	84.5	75.8	72.3	9.8
6	43.3	47.1	49.3	54.4	59.2	62.8	52.4	52.7	6.8
9	29.0	32.9	33.2	40.8	39.4	45.6	35.7	36.7	5.6
15	13.0	13.4	15.7	24.6	22.3	23.0	18.4	18.6	4.7
30	2.4	2.6	2.9	4.1	3.4	3.4	3.2	3.1	0.6

Table S14 (*R*)-MDMB-4en-PINACA percentage remaining (pHLM)

Time (min)	% parent compound remaining								
	1	2	3	4	5	6	7	Average	SD
0	100.0	100.0	100.0	100.0	100.0	100.0	100.0	100.0	-
3	61.1	64.2	64.3	71.2	69.2	81.2	67.6	68.4	6.6
6	43.1	46.9	46.5	50.4	50.5	55.9	43.9	48.2	4.5
9	29.0	32.0	30.3	32.7	34.5	38.6	29.1	32.3	3.4
15	11.1	13.1	12.1	15.9	16.9	18.6	14.1	14.6	2.7
30	1.6	2.3	2.0	3.2	2.8	3.2	2.4	2.5	0.6

Table S15 (*S*)-AB-FUBINACA percentage remaining (pHLM)

Time (min)	% parent compound remaining				
	1	2	3	Average	SD
0	100.0	100.0	100.0	100.0	-
3	88.5	80.9	92.6	87.3	5.9
6	83.5	81.6	96.7	87.3	8.2
9	87.1	78.7	89.3	85.0	5.6
15	84.9	81.6	86.0	84.2	2.3
30	74.8	77.9	86.0	79.6	5.7

Table S16 (*R*)-AB-FUBINACA percentage remaining (pHLM)

Time (min)	% parent compound remaining				
	1	2	3	Average	SD
0	100.0	100.0	100.0	100.0	-
3	88.7	90.6	89.2	89.5	1.0
6	85.0	84.4	102.3	90.5	10.2
9	94.7	88.3	82.3	88.4	6.2
15	80.5	83.6	84.6	82.9	2.2
30	79.7	82.8	89.2	83.9	4.9

Table S17 (*S*)-AMB-4en-PICA percentage remaining (pHLM)

Time (min)	% parent compound remaining											
	1	2	3	4	5	6	7	8	9	10	Average	SD
0	100.0	100.0	100.0	100.0	100.0	100.0	100.0	100.0	100.0	100.0	100.0	-
3	62.0	57.8	56.5	70.1	73.6	66.9	69.5	70.9	80.7	67.1	67.6	7.7
6	38.4	40.9	41.3	54.0	55.9	52.2	51.5	53.0	58.3	49.4	49.5	7.3
9	29.0	30.3	30.3	40.7	42.4	37.9	36.8	37.3	42.0	34.7	36.3	5.2
15	9.7	13.7	9.6	22.6	24.2	20.1	20.0	20.3	21.5	18.6	18.0	5.5
30	1.4	2.0	1.8	5.1	5.7	3.9	2.2	2.4	2.2	1.7	3.0	1.5

Table S18 (*R*)-AMB-4en-PICA percentage remaining (pHLM)

Time (min)	% parent compound remaining												
	1	2	3	4	5	6	7	8	9	10	11	Average	SD
0	100.0	100.0	100.0	100.0	100.0	100.0	100.0	100.0	100.0	100.0	100.0	100.0	-
3	49.8	52.4	48.3	45.7	51.2	63.9	61.7	60.0	67.7	63.3	57.3	56.4	7.8
6	32.0	32.1	29.5	22.0	38.0	42.0	43.2	37.8	41.2	44.1	35.2	36.2	7.1
9	19.4	17.1	18.8	14.3	21.9	28.6	29.1	23.3	27.2	26.5	21.8	22.6	5.2
15	6.0	6.5	6.0	4.4	10.0	13.8	14.1	10.7	12.5	11.8	9.1	9.6	3.6
30	0.6	0.4	0.6	0.5	2.5	4.4	4.3	1.1	1.2	0.5	0.5	1.6	1.6

Table S19 (*S*)-MDMB-FUBINACA percentage remaining (pHLM)

Time (min)	% parent compound remaining							
	1	2	3	4	5	6	Average	SD
0	100.0	100.0	100.0	100.0	100.0	100.0	100.0	-
3	62.1	84.4	74.0	68.0	63.8	69.0	70.2	8.1
6	63.6	75.6	56.2	73.6	56.0	60.1	64.2	8.6
9	45.8	64.9	55.8	66.7	48.2	53.2	55.8	8.5
15	18.6	39.6	39.9	46.8	30.2	30.7	34.3	9.9
30	13.4	19.1	16.7	19.0	15.0	14.9	16.4	2.3

Table S20 (*R*)-MDMB-FUBINACA percentage remaining (pHLM)

Time (min)	% parent compound remaining							
	1	2	3	4	5	6	Average	SD
0	100.0	100.0	100.0	100.0	100.0	100.0	100.0	-
3	77.4	71.6	86.0	93.0	68.0	67.7	77.3	10.3
6	65.4	81.1	69.8	82.6	75.4	76.0	75.0	6.6
9	65.4	62.5	66.7	77.7	81.7	67.0	70.2	7.7
15	51.3	57.5	56.8	62.0	60.6	54.9	57.2	3.9
30	27.4	34.5	36.9	34.8	35.6	30.6	33.3	3.6

Table S21 (*S*)-AB-CHMINACA percentage remaining (pHLM)

Time (min)	% parent compound remaining				
	1	2	3	Average	SD
0	100.0	100.0	100.0	100.0	-
3	73.2	77.8	77.5	76.2	2.6
6	69.8	77.8	82.4	76.7	6.4
9	67.0	71.0	73.7	70.6	3.3
15	56.4	63.3	65.3	61.7	4.7
30	43.4	45.7	48.5	45.8	2.6

Table S22 (*R*)-AB-CHMINACA percentage remaining (pHLM)

Time (min)	% parent compound remaining				
	1	2	3	Average	SD
0	100.0	100.0	100.0	100.0	-
3	84.2	83.1	84.8	84.0	0.9
6	85.7	83.1	84.4	84.4	1.3
9	81.1	81.0	84.0	82.1	1.7
15	72.4	77.9	70.5	73.6	3.9
30	58.7	61.0	57.4	59.0	1.8

Table S23 (*S*)-AMB-CHMICA percentage remaining (pHLM)

Time (min)	% parent compound remaining				
	1	2	3	Average	SD
0	100.0	100.0	100.0	100.0	-
3	56.1	54.1	47.8	52.7	4.3
6	39.2	38.7	27.4	35.1	6.7
9	26.8	27.6	21.4	25.3	3.4
15	14.0	13.8	1.9	9.9	6.9
30	3.4	3.3	0.7	2.5	1.5

Table S24 (*R*)-AMB-CHMICA percentage remaining (pHLM)

Time (min)	% parent compound remaining				
	1	2	3	Average	SD
0	100.0	100.0	100.0	100.0	-
3	86.9	63.7	68.3	73.0	12.3
6	57.0	50.7	53.1	53.6	3.2
9	54.2	44.7	43.3	47.4	5.9
15	34.7	28.2	28.2	30.4	3.7
30	12.8	10.5	10.7	11.3	1.3

Table S25 (*S*)-5F-AMB-PINACA percentage remaining (pHLM)

Time (min)	% parent compound remaining							
	1	2	3	4	5	6	Average	SD
0	100.0	100.0	100.0	100.0	100.0	100.0	100.0	-
3	10.0	12.5	11.1	12.0	10.0	8.0	10.6	1.6
6	0.0	0.0	3.7	4.0	3.3	0.0	1.8	2.0
9	0.0	0.0	0.0	0.0	0.0	0.0	0.0	0.0
15	0.0	0.0	0.0	0.0	0.0	0.0	0.0	0.0
30	0.0	0.0	0.0	0.0	0.0	0.0	0.0	0.0

Table S26 (*R*)-5F-AMB-PINACA percentage remaining (pHLM)

Time (min)	% parent compound remaining							
	1	2	3	4	5	6	Average	SD
0	100.0	100.0	100.0	100.0	100.0	100.0	100.0	-
3	62.3	60.8	60.0	64.7	66.7	70.0	64.1	3.8
6	46.4	46.2	52.3	52.5	53.5	55.0	51.0	3.7
9	35.5	37.1	38.1	41.7	38.0	37.9	38.0	2.0
15	20.2	19.9	21.9	21.6	22.5	24.3	21.7	1.6
30	6.6	6.5	7.1	7.2	6.2	7.1	6.8	0.4

Table S27 (*S*)-MDMB-4en-PICA percentage remaining (pHLM)

Time (min)	% parent compound remaining												
	1	2	3	4	5	6	7	8	9	10	11	Average	SD
0	100.0	100.0	100.0	100.0	100.0	100.0	100.0	100.0	100.0	100.0	100.0	100.0	-
3	68.5	75.0	73.4	73.0	82.6	82.0	84.0	75.1	78.4	85.1	75.0	77.5	5.3
6	58.1	58.6	56.5	62.1	68.7	67.3	71.6	60.7	60.2	65.9	59.2	62.6	4.9
9	60.9	43.3	44.9	44.2	56.5	55.8	59.6	48.7	44.5	51.2	43.1	50.2	6.9
15	21.3	24.2	22.2	23.4	37.0	36.6	38.0	32.0	25.5	28.6	24.4	28.5	6.3
30	3.5	2.9	3.5	3.1	10.7	9.5	13.2	5.0	3.4	3.6	3.1	5.6	3.7

Table S28 (*R*)-MDMB-4en-PICA percentage remaining (pHLM)

Time (min)	% parent compound remaining										
	1	2	3	4	5	6	7	8	9	Average	SD
0	100.0	100.0	100.0	100.0	100.0	100.0	100.0	100.0	100.0	100.0	-
3	56.1	57.9	65.9	68.5	75.4	70.5	72.2	75.3	67.6	67.7	6.9
6	36.0	41.8	48.2	49.0	55.7	51.7	49.5	51.8	45.1	47.7	5.9
9	23.8	27.4	34.1	32.6	38.5	34.2	32.1	34.0	28.9	31.7	4.4
15	7.6	9.2	16.5	14.9	18.5	15.1	14.6	15.1	13.0	13.8	3.4
30	0.5	0.7	3.6	2.7	3.4	1.0	1.1	1.0	0.9	1.7	1.2

Table S29 Intrinsic clearance (CL_{int}) with predicted *in vivo* hepatic clearance (CL_H) and hepatic extraction ratio (E_H) for pooled human liver microsome incubations (n≥3 ±SD) based on a microsomal scaling factor of 32 mg microsomal protein per gram liver [10]

Compound	Intrinsic clearance, CL_{int} (mL min^{-1} kg^{-1})	Predicted *in vivo* hepatic clearance, CL_H (mL min^{-1} kg^{-1})	Hepatic extraction ratio, E_H	n
(*S*)-AMB-FUBINACA (**1**)	1794 ±58.4	12.99 ±0.16	0.62 ±0.008	3
(*R*)-AMB-FUBINACA (**1**)	195 ±16.3	-	-	6
(*S*)-5F-AMB-PINACA (**10**)	1229 ±94.9	16.21 ±0.27	0.77 ±0.013	6
(*R*)-5F-AMB-PINACA (**10**)	175 ±11.6	-	-	6
(*S*)-AMB-CHMICA (**9**)	282 ±56.6	2.91 ±0.50	0.14 ±0.024	3
(*R*)-AMB-CHMICA (**9**)	138 ±15.5	-	-	3
(*S*)-AMB-4en-PICA (**7**)	194 ±34.5	6.86 ±0.80	0.33 ±0.038	10
(*R*)-AMB-4en-PICA (**7**)	286 ±56.2	9.28 ±0.98	0.44 ±0.047	11
(*S*)-MDMB-4en-PINACA (**5**)	186 ±30.0	1.71 ±0.25	0.08 ±0.012	7
(*R*)-MDMB-4en-PINACA (**5**)	208 ±21.1	3.32 ±0.29	0.16 ±0.014	7
(*S*)-5F-MDMB-PINACA (**2**)	171 ±7.80	3.18 ±0.12	0.15 ±0.006	5
(*R*)-5F-MDMB-PINACA (**2**)	145 ±19.3	4.53 ±0.48	0.22 ±0.023	5
(*S*)-5F-MDMB-PICA (**3**)	128 ±22.5	5.75 ±0.71	0.27 ±0.034	3
(*R*)-5F-MDMB-PICA (**3**)	108 ±1.13	5.09 ±0.04	0.24 ±0.002	3
(*S*)-MDMB-4en-PICA (**12**)	137 ±19.7	3.88 ±0.47	0.18 ±0.022	11
(*R*)-MDMB-4en-PICA (**12**)	213 ±32.5	7.32 ±0.70	0.35 ±0.033	9
(*S*)-4F-MDMB-BINACA (**4**)	118 ±20.6	5.33 ±0.72	0.25 ±0.034	3
(*R*)-4F-MDMB-BINACA (**4**)	79 ±3.82	6.20 ±0.21	0.30 ±0.010	3
(*S*)-MDMB-FUBINACA (**6**)	111 ±24.5	0.54 ±0.12	0.03 ±0.006	6
(*R*)-MDMB-FUBINACA (**6**)	58.2 ±7.5	-	-	6
(*S*)-AB-CHMINACA (**11**)	42.1 ±4.48	1.12 ±0.11	0.05 ±0.005	3
(*R*)-AB-CHMINACA (**11**)	26.9 ±2.98	-	-	3
(*S*)-AB-FUBINACA (**8**)	10.0 ±2.59	0.21 ±0.05	0.01 ±0.003	3
(*R*)-AB-FUBINACA (**8**)	8.4 ±2.47	-	-	3

Table S30 Intrinsic clearance (CL_{int}) with predicted *in vivo* hepatic clearance (CL_H) and hepatic extraction ratio (E_H) for pooled human liver microsome incubations (n≥3 ±SD) based on a microsomal scaling factor of 45 mg microsomal protein per gram liver [11]

Compound	Intrinsic clearance, CL_{int} (mL min^{-1} kg^{-1})	Predicted *in vivo* hepatic clearance, CL_H (mL min^{-1} kg^{-1})	Hepatic extraction ratio, E_H	n
(*S*)-AMB-FUBINACA (**1**)	2523 ±82.2	14.60 ±0.14	0.70 ±0.007	3
(*R*)-AMB-FUBINACA (**1**)	274 ±23.0	-	-	6
(*S*)-5F-AMB-PINACA (**10**)	1728 ±133.4	17.35 ±0.22	0.83 ±0.011	6
(*R*)-5F-AMB-PINACA (**10**)	247 ±16.4	-	-	6
(*S*)-AMB-CHMICA (**9**)	397 ±79.7	3.87 ±0.62	0.18 ±0.030	3
(*R*)-AMB-CHMICA (**9**)	193 ±21.8	-	-	3
(*S*)-AMB-4en-PICA (**7**)	273 ±48.5	8.51 ±0.87	0.41 ±0.041	10
(*R*)-AMB-4en-PICA (**7**)	402 ±79.0	11.05 ±0.98	0.53 ±0.047	11
(*S*)-MDMB-4en-PINACA (**5**)	262 ±42.3	2.32 ±0.33	0.11 ±0.016	7
(*R*)-MDMB-4en-PINACA (**5**)	292 ±29.7	4.38 ±0.36	0.21 ±0.017	7
(*S*)-5F-MDMB-PINACA (**2**)	240 ±11.0	4.22 ±0.15	0.20 ±0.007	5
(*R*)-5F-MDMB-PINACA (**2**)	204 ±27.2	5.85 ±0.57	0.28 ±0.027	5
(*S*)-5F-MDMB-PICA (**3**)	181 ±31.6	7.27 ±0.81	0.35 ±0.038	3
(*R*)-5F-MDMB-PICA (**3**)	152 ±1.59	6.51 ±0.05	0.31 ±0.002	3
(*S*)-MDMB-4en-PICA (**12**)	192 ±27.8	5.07 ±0.57	0.24 ±0.027	11
(*R*)-MDMB-4en-PICA (**12**)	300 ±45.7	9.01 ±0.74	0.43 ±0.035	9
(*S*)-4F-MDMB-BINACA (**4**)	165 ±28.9	6.78 ±0.83	0.32 ±0.040	3
(*R*)-4F-MDMB-BINACA (**4**)	112 ±5.37	7.78 ±0.23	0.37 ±0.011	3
(*S*)-MDMB-FUBINACA (**6**)	156 ±34.4	0.75 ±0.16	0.04 ±0.008	6
(*R*)-MDMB-FUBINACA (**6**)	81.8 ±10.5	-	-	6
(*S*)-AB-CHMINACA (**11**)	59.2 ±6.30	1.54 ±0.15	0.08 ±0.007	3
(*R*)-AB-CHMINACA (**11**)	37.8 ±4.19	-	-	3
(*S*)-AB-FUBINACA (**8**)	14.1 ±3.64	0.29 ±0.07	0.01 ±0.004	3
(*R*)-AB-FUBINACA (**8**)	11.8 ±3.48	-	-	3

References

10. Barter, Z.E.; Bayliss, M.K.; Beaune, P.H.; Boobis, A.R.; Carlile, D.J.; Edwards, R.J.; Houston, J.B.; Lake, B.G.; Lipscomb, J.C.; Pelkonen, O.R.; Tucker, G.T.; Rostami-Hodjegan, A. Scaling factors for the extrapolation of in vivo metabolic drug clearance from in vitro data: reaching a consensus on values of human microsomal protein and hepatocellularity per gram of liver. *Curr. Drug. Metab.* **2007**, *8*, 33-45. doi: 10.2174/138920007779315053.

11. Watanabe, S.; Kuzhiumparambil, U.; Fu, S. In vitro metabolism of synthetic cannabinoid AM1220 by human liver microsomes and Cunninghamella elegans using liquid chromatography coupled with high resolution mass spectrometry. *Forensic Toxicol.* **2018**, *36*, 435-446. doi: 10.1007/s11419-018-0424-y.

Section S7

Intrinsic clearance data – pooled human cryopreserved hepatocyte incubations

The percentage of parent compound remaining, based on peak area ratio of analyte/internal standard (donepezil) in cryopreserved human hepatocyte (HHeps) incubations is detailed below in figures S34-49, with enantiomers of each compound overlaid for comparison. Data for each replicate is provided in tables S31-57. Intrinsic clearance and predicted *in vivo* data calculated using an alternative hepatocyte cell density found in the literature – $99x10^6$ hepatocytes per gram of liver [10] – is provided in table S58.

Figure S34 Verapamil (control) clearance (pHHeps), 60 minutes, n=8, error bars represent ± standard deviation

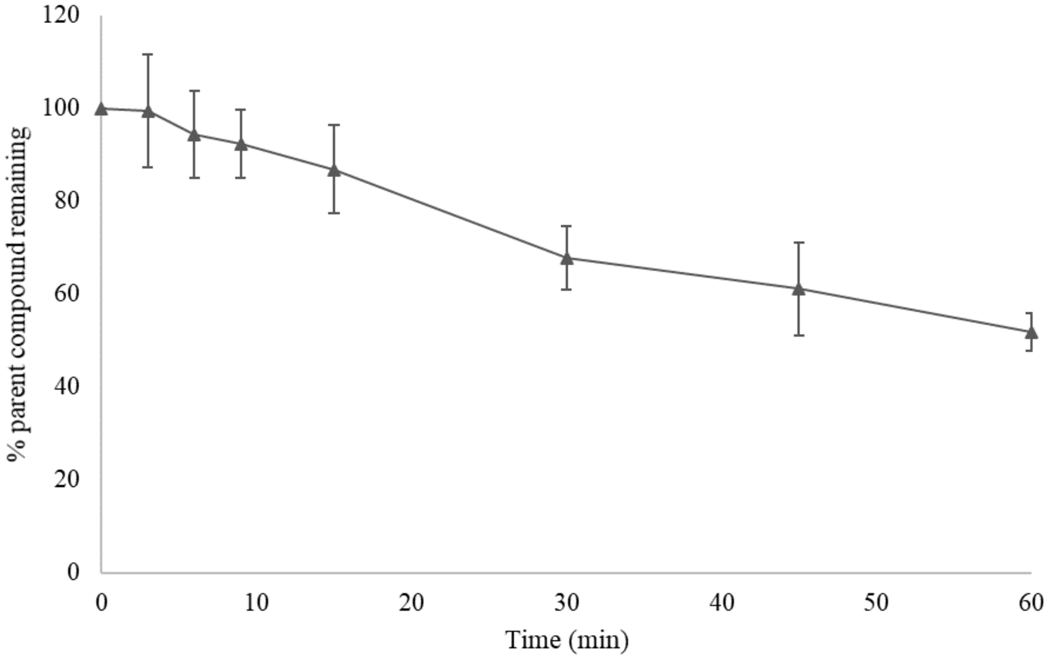

Figure S35 7-ethoxycoumarin (control) clearance (pHHeps), 60 minutes, n=8, error bars represent ± standard deviation

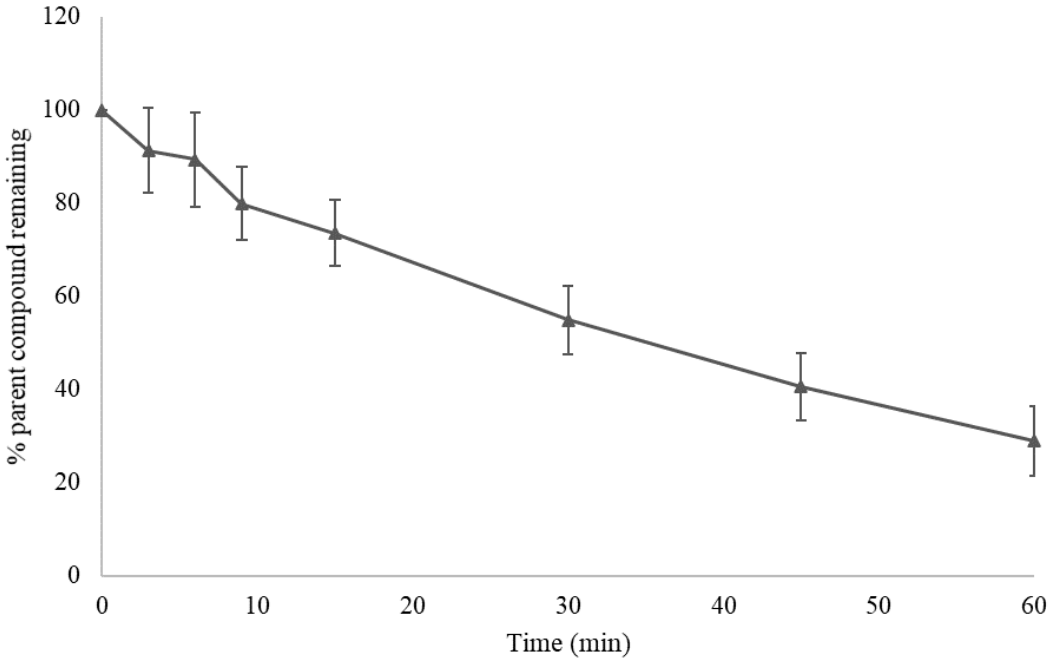

Figure S36 7-hydroxycoumarin (control) clearance (pHHeps), 60 minutes, n=7, error bars represent ± standard deviation

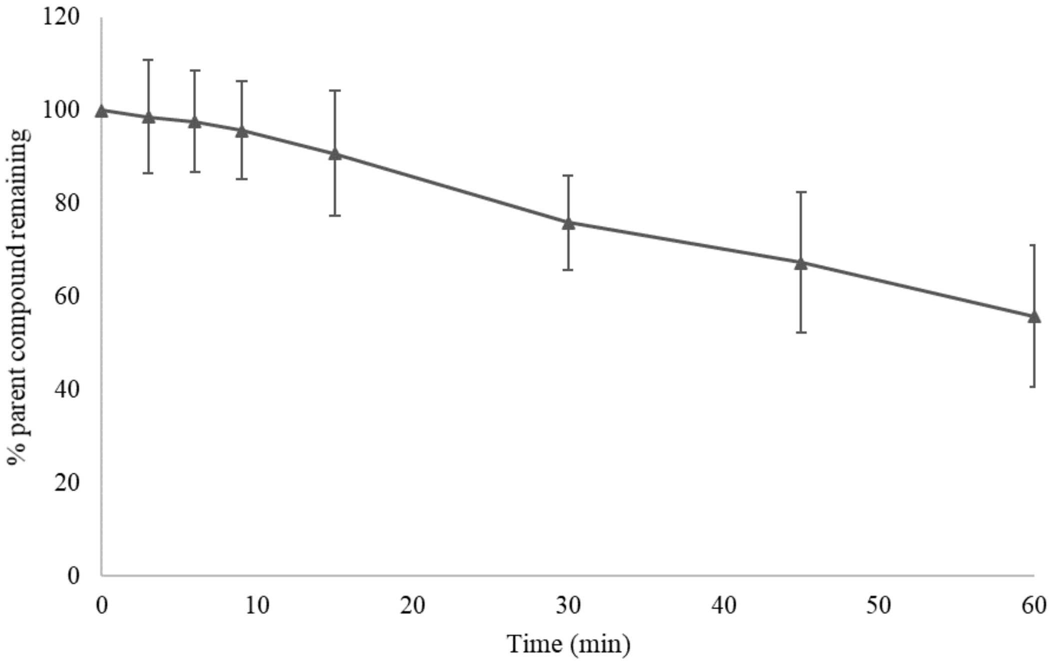

Figure S37 AMB-FUBINACA clearance (pHHeps), 60 minutes, n=8, error bars represent ± standard deviation

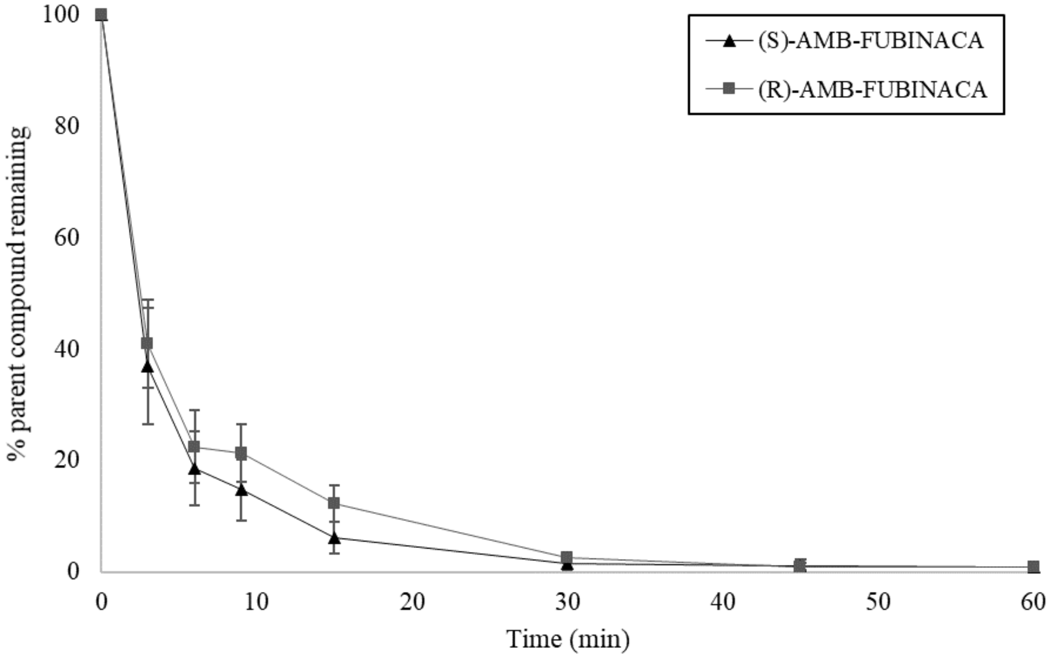

Figure S38 5F-MDMB-PINACA clearance (pHHeps lot HUE50-N), 60 minutes, n=8 ((*S*)-); n=6 ((*R*)-), error bars represent ± standard deviation

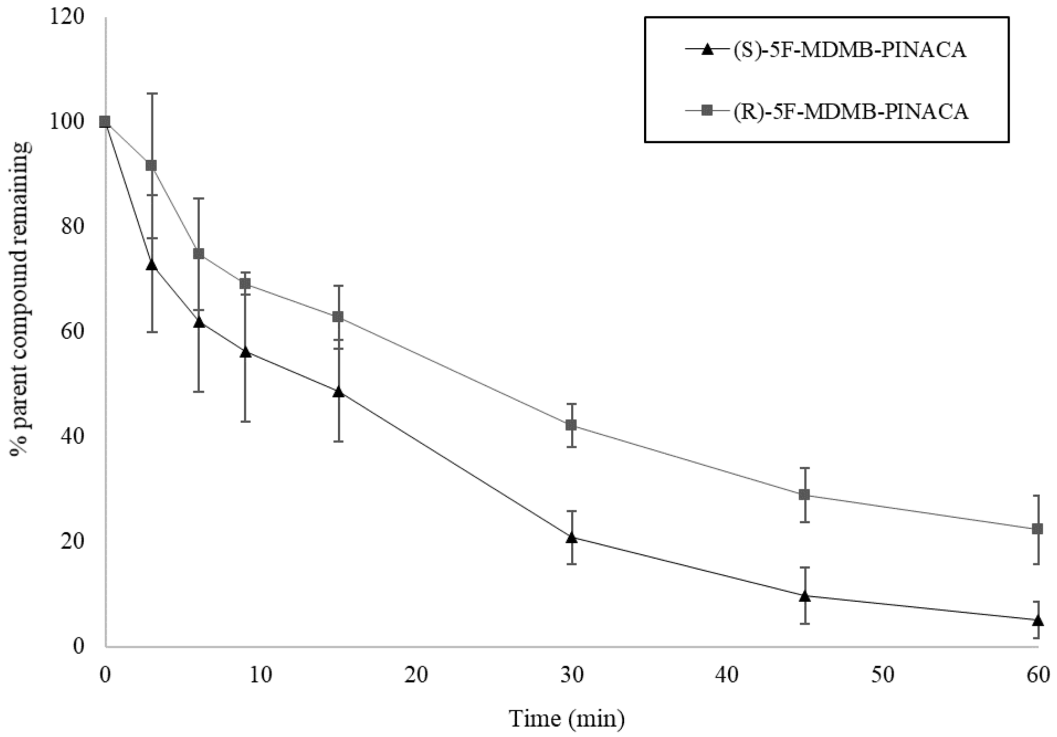

Figure S39 5F-MDMB-PINACA clearance (pHHeps lot HUE50-P), 60 minutes, n=4, error bars represent ± standard deviation

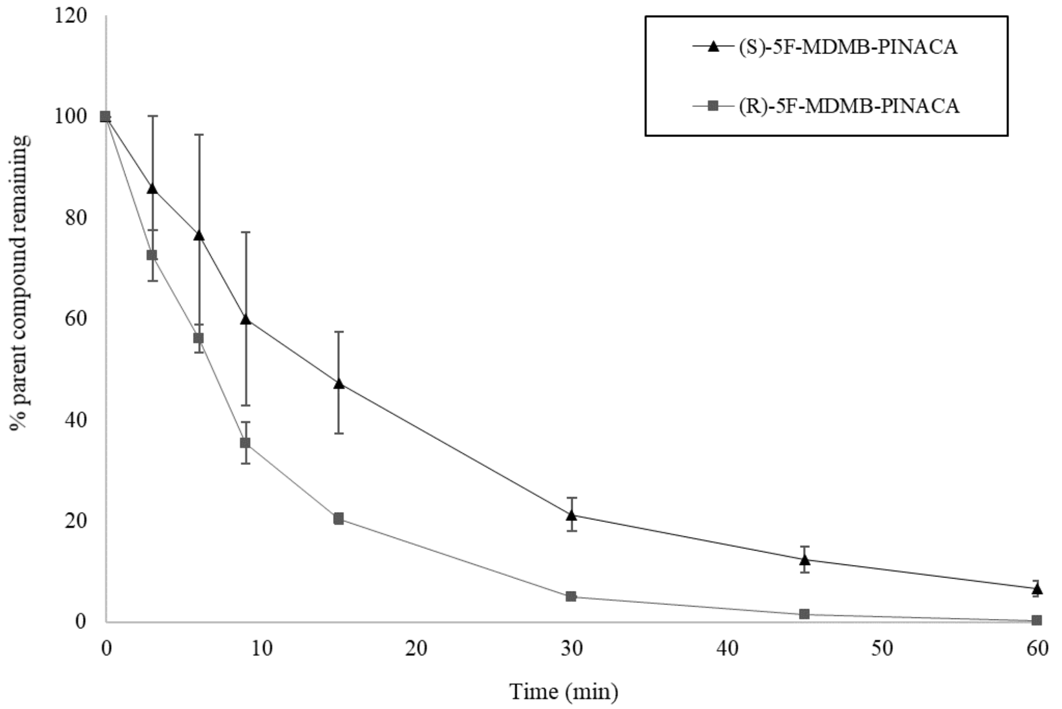

Figure S40 5F-MDMB-PICA clearance (pHHeps), 60 minutes, n=3, error bars represent ± standard deviation

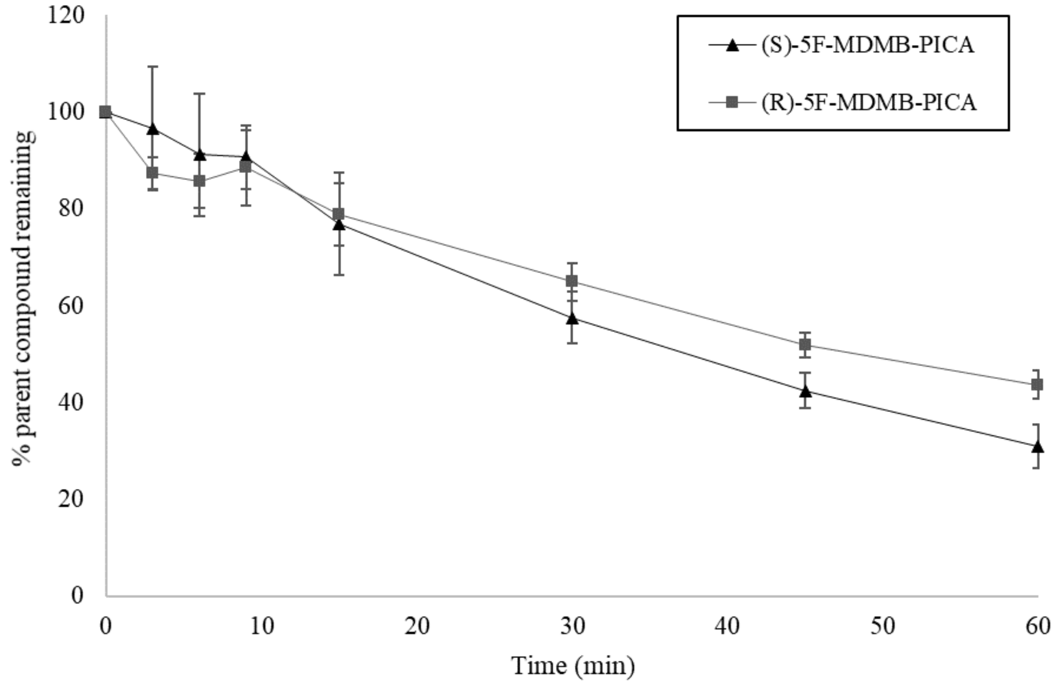

Figure S41 4F-MDMB-BINACA clearance (pHHeps), 60 minutes, n=3 ((*S*)-); n=4 ((*R*)-), error bars represent ± standard deviation

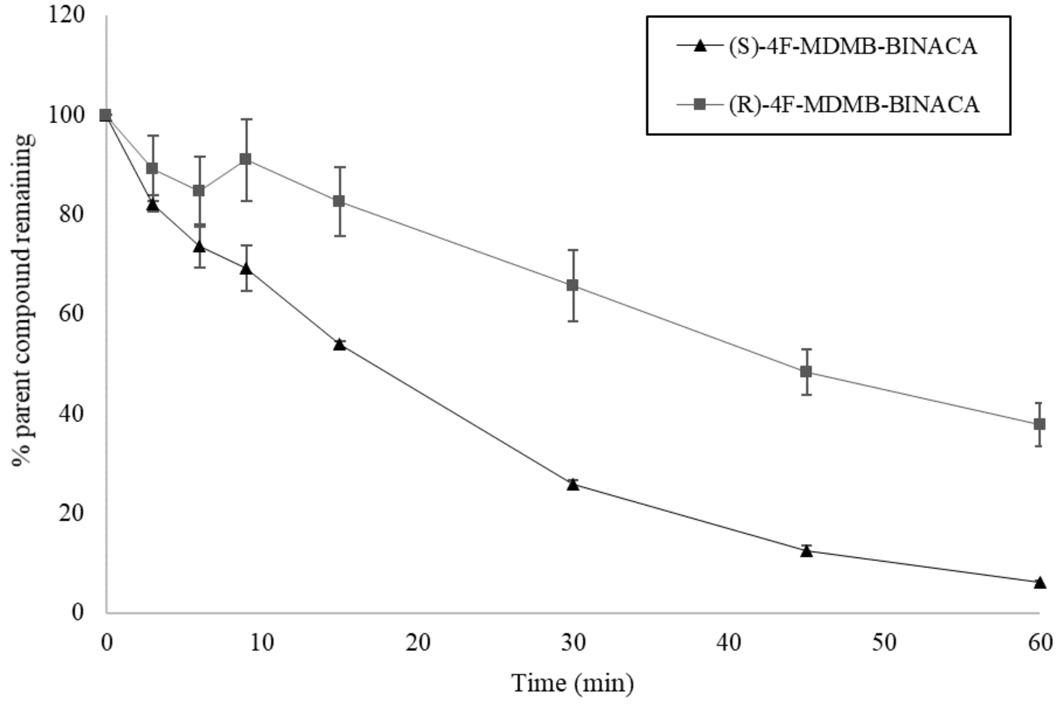

Figure S42 MDMB-4en-PINACA clearance (pHHeps), 60 minutes, n=7, error bars represent ± standard deviation

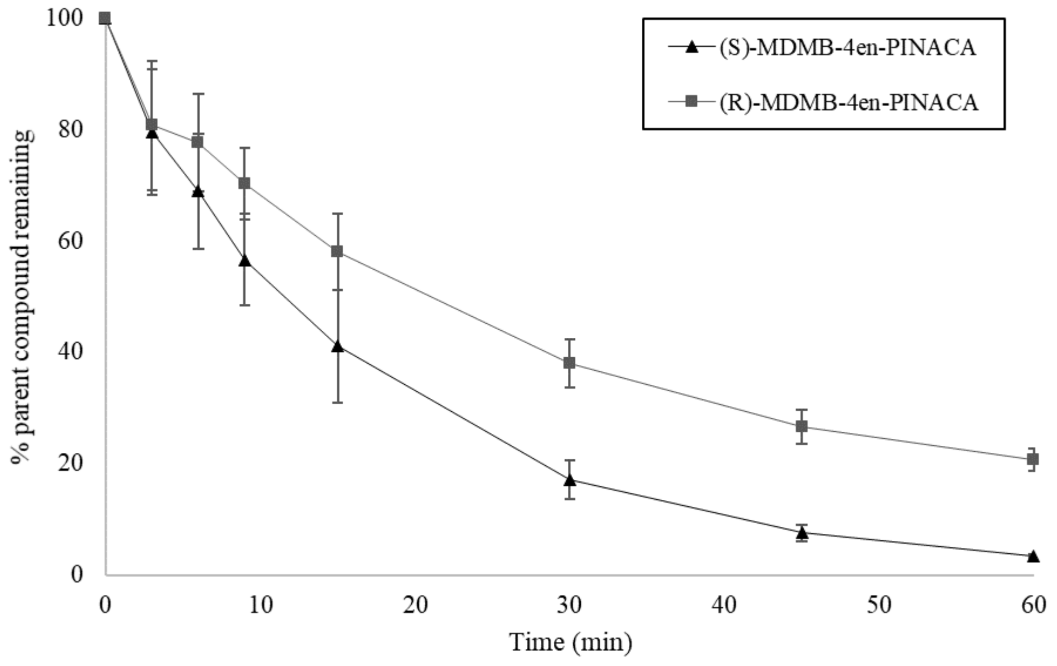

Figure S43 AB-FUBINACA clearance (pHHeps), 60 minutes, n=8, error bars represent ± standard deviation

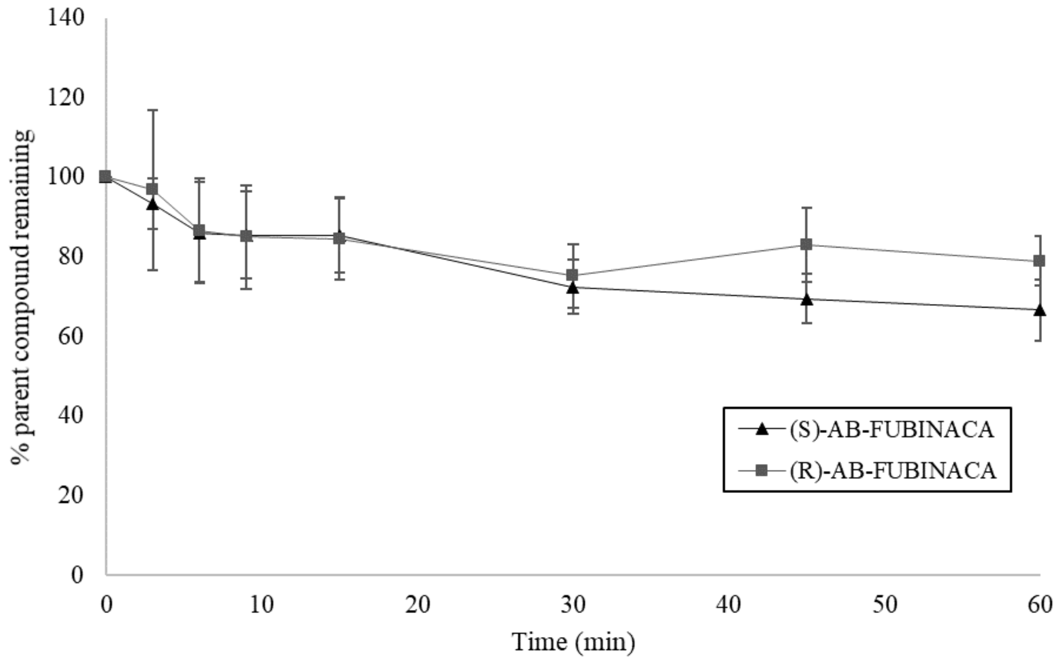

Figure S44 AMB-4en-PICA clearance (pHHeps), 60 minutes, n=8, error bars represent ± standard deviation

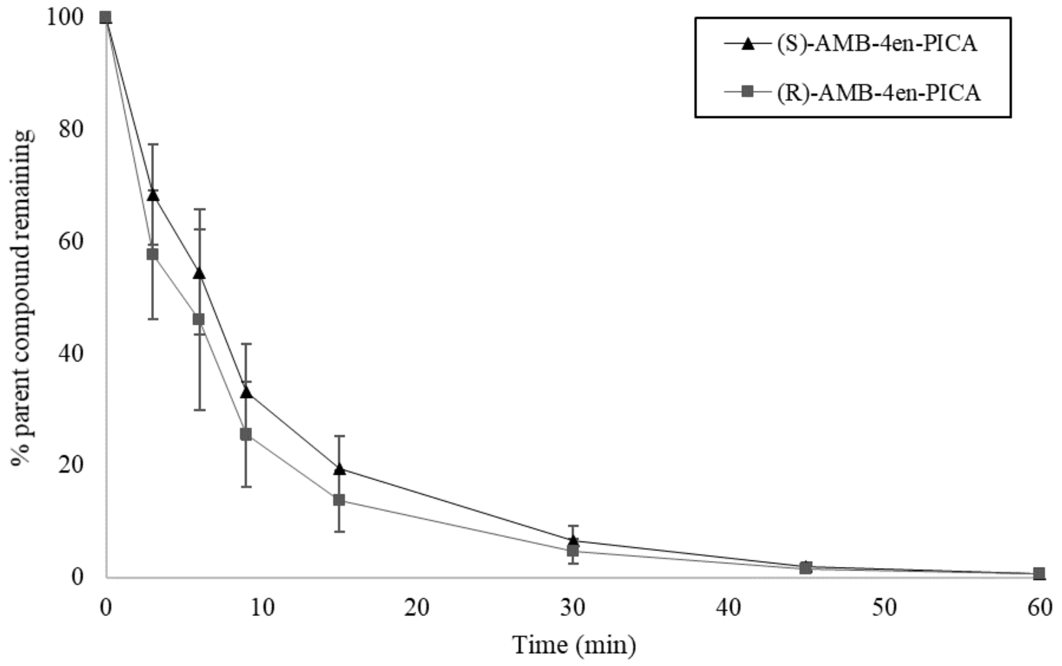

Figure S45 MDMB-FUBINACA clearance (pHHeps), 60 minutes, n=3, error bars represent ± standard deviation

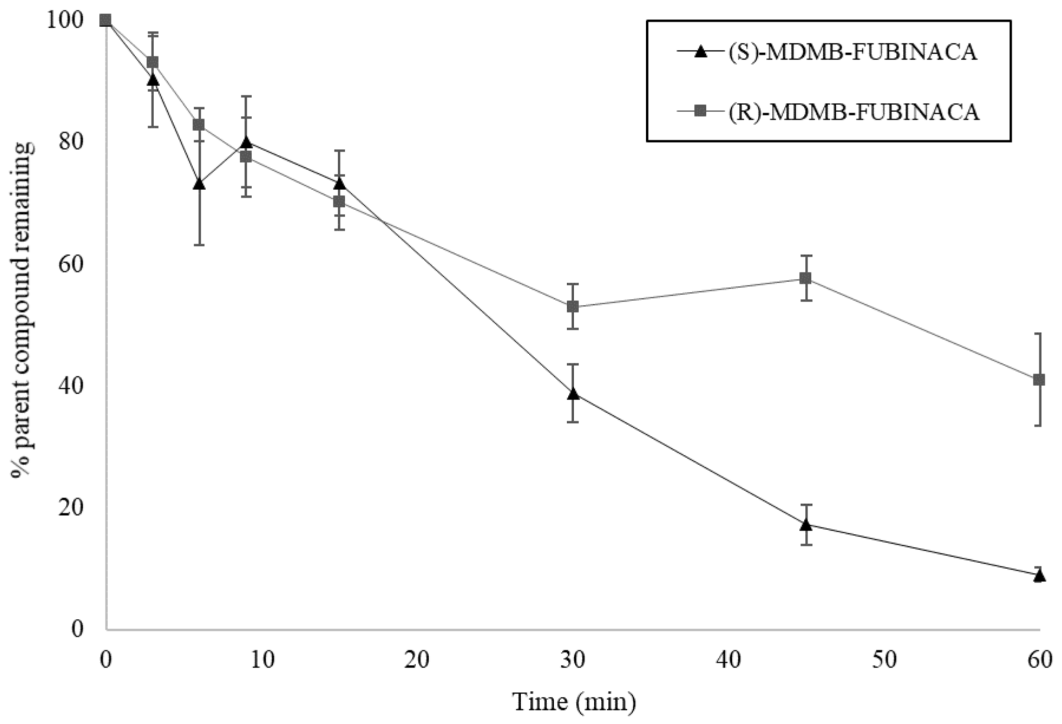

Figure S46 AB-CHMINACA clearance (pHHeps), 60 minutes, n=6 ((*S*)-); n=5 ((*R*)-), error bars represent ± standard deviation

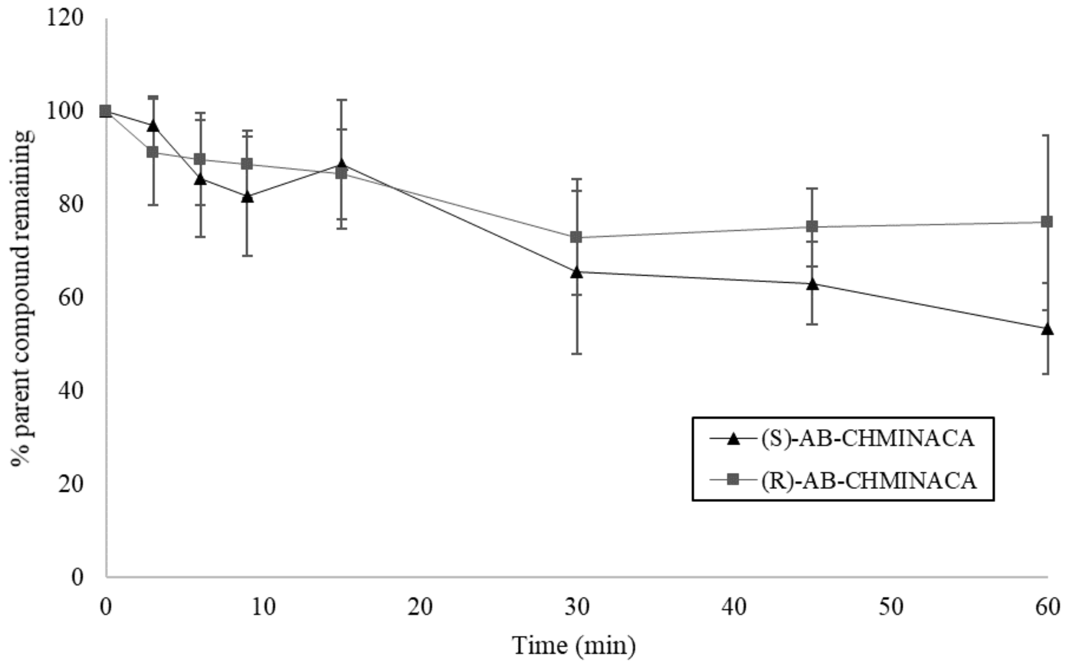

Figure S47 AMB-CHMICA clearance (pHHeps), 60 minutes, n=3, error bars represent ± standard deviation

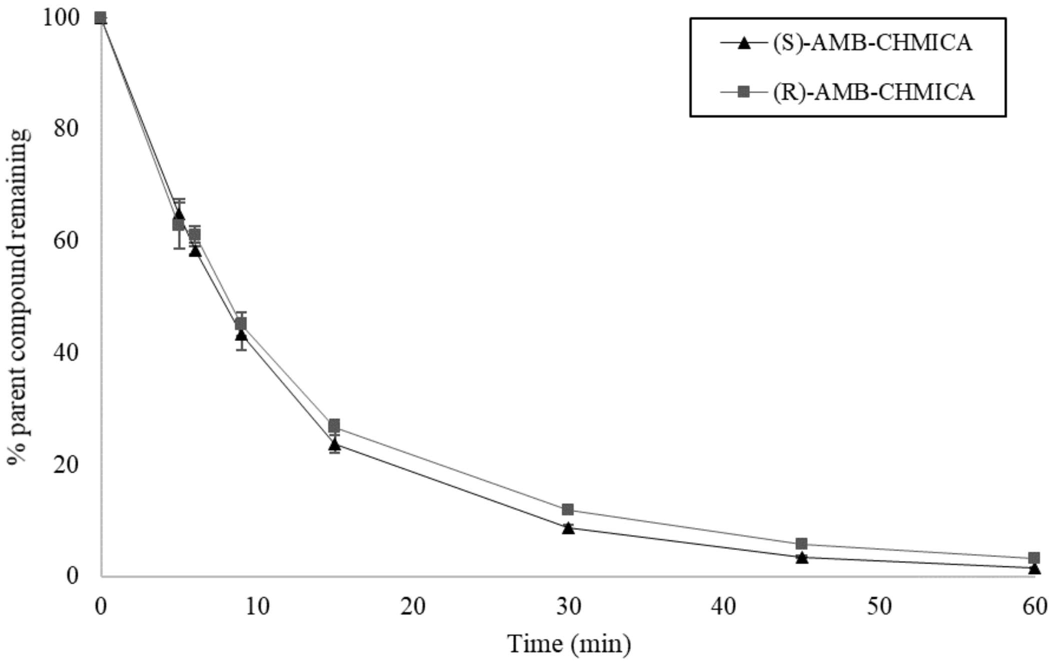

Figure S48 5F-AMB-PINACA clearance (pHHeps), 60 minutes, n=3, error bars represent ± standard deviation

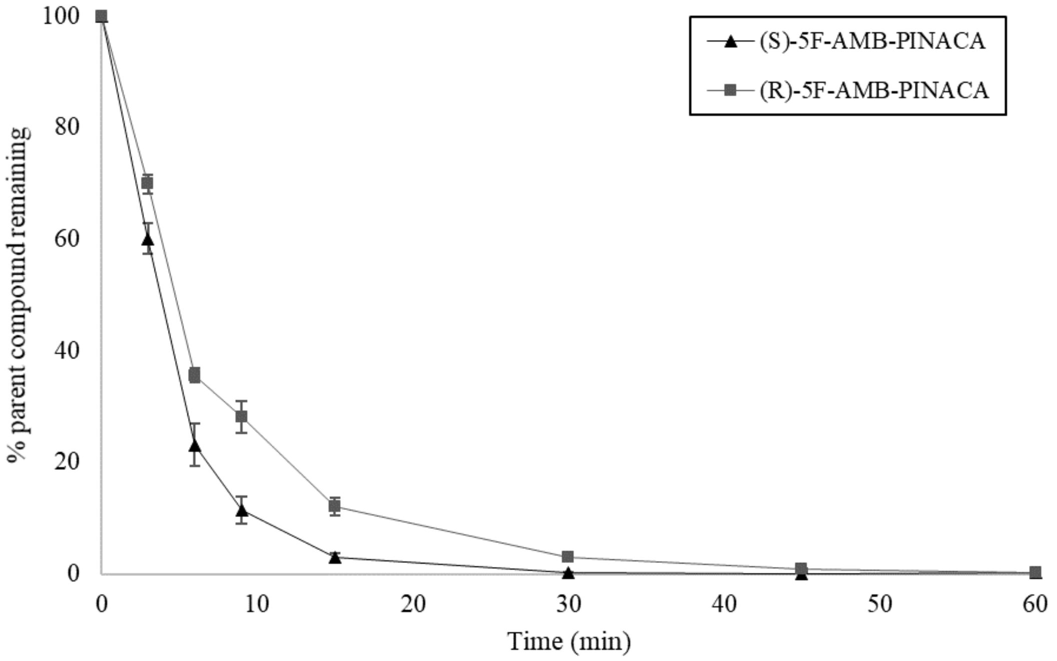

Figure S49 MDMB-4en-PICA clearance (pHHeps), 60 minutes, n=7, error bars represent ± standard deviation

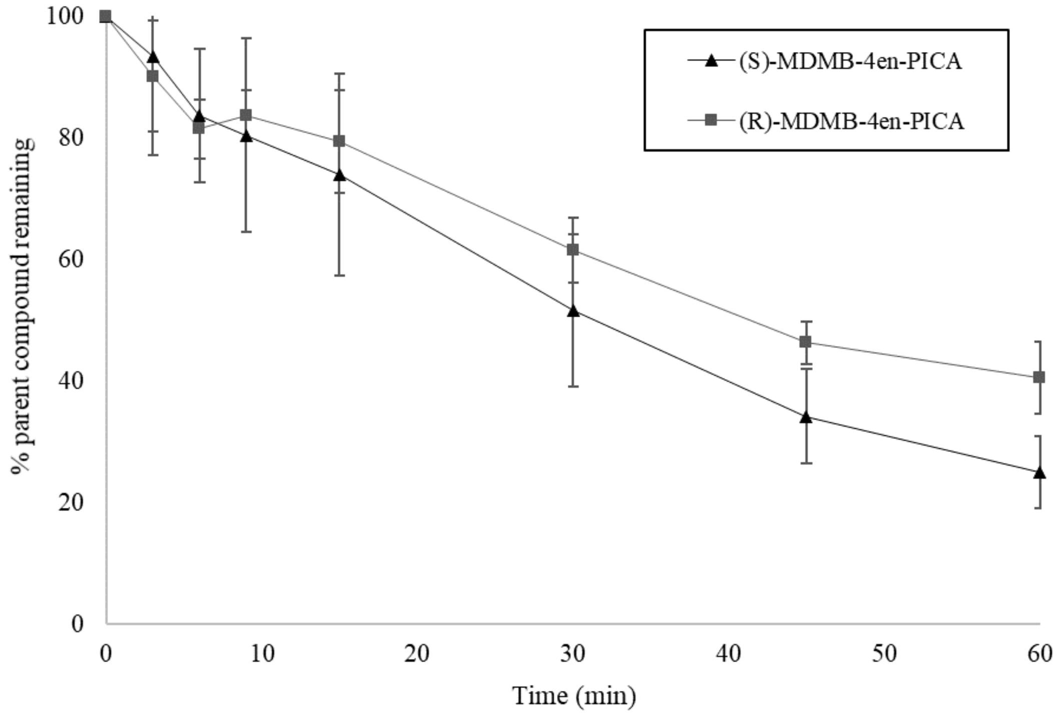

Table S31 Verapamil (control) percentage remaining (pHHeps)

Time (min)	% parent compound remaining 1	2	3	4	5	6	7	8	Average	SD
0	100.0	100.0	100.0	100.0	100.0	100.0	100.0	100.0	100.0	-
3	106.7	95.7	94.3	109.5	121.4	83.9	89.5	95.0	99.5	12.2
6	95.3	84.2	90.2	100.0	110.7	85.5	87.6	102.3	94.5	9.3
9	106.2	84.1	90.2	91.6	100.6	91.7	87.2	87.5	92.4	7.4
15	95.8	84.4	99.2	92.7	90.5	71.3	76.5	85.1	87.0	9.5
30	75.6	65.9	74.5	56.4	72.0	63.6	62.6	72.9	67.9	6.8
45	71.4	58.9	80.4	62.0	55.9	49.8	55.2	56.1	61.2	10.0
60	59.7	49.6	52.6	52.2	48.3	55.2	48.0	49.5	51.9	4.0

Table S32 7-ethoxycoumarin (control) percentage remaining (pHHeps)

Time (min)	% parent compound remaining 1	2	3	4	5	6	7	8	Average	SD
0	100.0	100.0	100.0	100.0	100.0	100.0	100.0	100.0	100.0	-
3	89.8	85.4	108.1	87.8	79.3	85.6	97.8	97.3	91.4	9.2
6	96.4	76.3	96.6	87.8	79.3	80.1	105.3	93.2	89.4	10.2
9	87.2	80.0	85.9	63.3	75.7	78.4	86.0	83.5	80.0	7.9
15	63.8	74.8	81.6	66.1	72.5	69.5	84.4	76.5	73.7	7.2
30	55.1	66.5	56.4	46.1	51.3	53.5	63.6	46.9	54.9	7.3
45	39.6	47.0	52.6	32.7	38.3	39.7	44.8	30.6	40.7	7.3
60	31.9	32.2	40.2	19.1	27.7	33.6	28.9	18.1	29.0	7.4

Table S33 7-hydroxycoumarin (control) percentage remaining (pHHeps)

Time (min)	% parent compound remaining 1	2	3	4	5	6	7	Average	SD
0	100.0	100.0	100.0	100.0	100.0	100.0	100.0	100.0	-
3	78.5	118.0	106.0	102.1	94.1	93.8	97.7	98.6	12.2
6	76.7	105.9	100.0	100.4	91.7	110.4	97.7	97.5	10.9
9	77.3	103.6	95.3	111.0	90.9	98.2	93.3	95.7	10.6
15	70.1	94.6	89.3	112.1	78.8	96.0	94.4	90.8	13.4
30	61.8	80.2	74.6	91.0	64.4	80.4	78.5	75.8	10.0
45	51.3	89.6	61.7	84.9	51.5	62.9	68.6	67.2	15.1
60	41.2	78.8	48.0	75.9	45.4	47.3	54.1	55.8	15.2

Table S34 (*S*)-AMB-FUBINACA percentage remaining (pHHeps)

Time (min)	% parent compound remaining									
	1	2	3	4	5	6	7	8	Average	SD
0	100.0	100.0	100.0	100.0	100.0	100.0	100.0	100.0	100.0	-
3	35.8	33.9	41.7	28.3	39.2	21.0	39.7	56.6	37.0	10.4
6	15.8	8.7	10.7	18.5	22.4	24.6	20.9	27.7	18.7	6.6
9	22.4	8.2	13.3	9.1	13.1	19.5	11.4	20.7	14.7	5.4
15	11.8	3.1	4.3	3.3	5.7	6.6	6.5	8.3	6.2	2.9
30	1.6	0.4	0.4	0.7	1.6	0.6	2.9	3.1	1.4	1.1
45	0.6	0.2	0.1	0.3	0.9	0.8	2.9	2.9	1.1	1.2
60	0.2	0.1	0.1	1.0	0.2	0.4	2.7	2.1	0.8	1.0

Table S35 (*R*)-AMB-FUBINACA percentage remaining (pHHeps)

Time (min)	% parent compound remaining									
	1	2	3	4	5	6	7	8	Average	SD
0	100.0	100.0	100.0	100.0	100.0	100.0	100.0	100.0	100.0	-
3	50.9	47.3	45.8	34.3	35.4	48.3	29.5	37.0	41.1	7.9
6	15.2	18.7	13.9	25.3	25.7	31.7	19.7	29.7	22.5	6.6
9	17.0	23.2	19.4	23.2	27.3	29.4	15.3	16.4	21.4	5.2
15	9.7	13.9	10.4	13.6	16.8	16.3	7.7	9.9	12.3	3.3
30	1.7	2.8	1.9	2.4	3.6	3.3	2.5	3.1	2.7	0.6
45	0.4	0.3	0.2	0.9	1.2	0.8	1.8	1.8	0.9	0.6
60	0.2	0.3	0.1	0.2	0.5	0.9	2.4	2.1	0.8	0.9

Table S36 (*S*)-5F-MDMB-PINACA percentage remaining (pHHeps), replicates 1-8 from lot HUE50-N; 9-12 from HUE50-P

Time (min)	% parent compound remaining													
	1	2	3	4	5	6	7	8	9	10	11	12	Average	SD
0	100.0	100.0	100.0	100.0	100.0	100.0	100.0	100.0	100.0	100.0	100.0	100.0	100.0	-
3	81.9	68.9	66.2	89.2	90.1	69.8	65.0	52.5	98.4	95.0	83.4	67.2	77.3	14.3
6	40.3	50.0	60.6	83.8	69.5	71.0	60.9	59.9	101.4	82.1	67.9	55.3	66.9	16.5
9	52.7	52.7	41.6	77.0	75.5	53.9	53.6	43.0	79.2	67.5	54.6	39.3	57.5	14.0
15	44.3	42.5	39.2	60.4	65.6	51.8	44.8	41.6	60.0	50.4	37.4	41.6	48.3	9.3
30	25.5	18.2	16.8	29.4	24.9	19.9	17.3	15.3	24.1	24.3	19.3	17.7	21.1	4.4
45	6.8	4.5	5.3	18.2	17.5	10.4	7.8	7.4	14.3	14.8	11.1	9.5	10.6	4.6
60	4.1	2.1	3.8	10.1	11.2	2.8	3.6	3.3	8.6	7.3	6.0	4.8	5.6	3.0

Table S37 (*R*)-5F-MDMB-PINACA percentage remaining (pHHeps), replicates 1-6 from lot HUE50-N; 7-10 from HUE50-P

Time (min)	% parent compound remaining											
	1	2	3	4	5	6	7	8	9	10	Average	SD
0	100.0	100.0	100.0	100.0	100.0	100.0	100.0	100.0	100.0	100.0	100.0	-
3	90.4	77.4	80.7	103.2	85.7	113.0	77.5	69.9	76.0	67.0	84.1	14.5
6	70.0	79.2	94.5	70.6	69.4	65.0	59.0	52.4	56.9	56.4	67.3	12.6
9	68.5	68.1	69.2	67.4	73.5	68.6	29.8	37.9	38.8	35.6	55.7	17.6
15	63.9	54.8	57.8	67.0	61.6	71.3	21.0	21.2	19.0	20.7	45.8	22.3
30	42.7	37.6	40.8	41.0	40.8	49.8	5.0	5.0	5.1	5.1	27.3	19.4
45	28.2	21.5	24.5	32.2	35.3	31.5	1.8	1.4	1.3	1.4	17.9	14.7
60	18.4	14.2	18.2	23.7	28.2	31.3	0.5	0.4	0.4	0.4	13.6	12.3

Table S38 (*S*)-5F-MDMB-PICA percentage remaining (pHHeps)

Time (min)	% parent compound remaining				
	1	2	3	Average	SD
0	100.0	100.0	100.0	100.0	-
3	111.3	88.1	90.6	96.7	12.7
6	105.9	83.4	84.3	91.2	12.7
9	98.2	85.8	88.3	90.7	6.6
15	89.2	72.0	69.8	77.0	10.6
30	63.7	54.0	54.9	57.6	5.4
45	46.6	39.6	41.5	42.5	3.6
60	36.1	28.6	28.1	30.9	4.5

Table S39 (*R*)-5F-MDMB-PICA percentage remaining (pHHeps)

Time (min)	% parent compound remaining				
	1	2	3	Average	SD
0	100.0	100.0	100.0	100.0	-
3	89.6	89.1	83.6	87.4	3.3
6	88.9	89.1	79.4	85.8	5.6
9	96.9	87.3	81.5	88.6	7.8
15	85.6	78.1	72.8	78.8	6.4
30	68.6	65.1	61.0	64.9	3.8
45	53.4	53.3	48.8	51.8	2.6
60	46.9	43.1	41.1	43.7	2.9

<u>Table S40</u> <u>(*S*)-4F-MDMB-BINACA percentage remaining (pHHeps)</u>

Time (min)	% parent compound remaining				
	1	2	3	Average	SD
0	100.0	100.0	100.0	100.0	-
3	83.5	82.6	80.6	82.3	1.5
6	71.6	70.9	78.6	73.7	4.3
9	70.3	64.2	73.1	69.2	4.5
15	54.8	53.5	53.4	53.9	0.8
30	26.7	24.8	25.9	25.8	1.0
45	13.4	11.4	12.7	12.5	1.0
60	6.6	5.7	6.2	6.2	0.5

<u>Table S41</u> <u>(*R*)-4F-MDMB-BINACA percentage remaining (pHHeps)</u>

Time (min)	% parent compound remaining					
	1	2	3	4	Average	SD
0	100.0	100.0	100.0	100.0	100.0	-
3	88.0	98.6	87.0	83.3	89.2	6.6
6	74.9	84.5	90.6	88.5	84.6	7.0
9	82.7	99.7	96.3	85.1	91.0	8.3
15	74.7	90.7	85.0	79.9	82.6	6.9
30	64.8	74.8	66.2	57.1	65.7	7.3
45	47.6	54.4	48.7	43.3	48.5	4.6
60	39.1	42.3	38.4	31.9	37.9	4.3

Table S42 (*S*)-MDMB-4en-PINACA percentage remaining (pHHeps)

Time (min)	% parent compound remaining								
	1	2	3	4	5	6	7	Average	SD
0	100.0	100.0	100.0	100.0	100.0	100.0	100.0	100.0	-
3	79.8	69.5	73.8	76.4	102.9	83.4	71.8	79.7	11.3
6	63.7	56.2	60.3	68.9	87.1	73.5	73.2	69.0	10.3
9	53.0	45.8	49.2	56.2	69.3	62.5	60.8	56.7	8.2
15	33.3	28.7	30.5	44.9	55.2	47.9	47.1	41.1	10.2
30	15.4	12.9	13.1	17.1	19.8	20.0	21.4	17.1	3.4
45	7.3	6.0	5.8	6.9	9.2	8.4	9.2	7.6	1.4
60	3.8	3.0	3.0	3.1	3.5	3.9	3.5	3.4	0.4

Table S43 (*R*)-MDMB-4en-PINACA percentage remaining (pHHeps)

Time (min)	% parent compound remaining								
	1	2	3	4	5	6	7	Average	SD
0	100.0	100.0	100.0	100.0	100.0	100.0	100.0	100.0	-
3	83.0	80.0	77.3	89.8	81.7	95.5	58.5	80.8	11.6
6	74.6	67.5	66.1	84.4	83.0	89.7	78.3	77.7	8.8
9	67.5	67.0	59.6	77.3	74.4	77.5	69.0	70.3	6.5
15	53.5	52.6	48.4	66.0	66.4	61.0	58.8	58.1	6.9
30	37.7	35.4	30.8	40.2	36.9	44.4	40.9	38.0	4.4
45	23.5	24.9	22.8	29.4	26.4	29.2	30.1	26.6	3.0
60	19.3	19.9	18.1	23.5	22.8	22.0	19.8	20.8	2.0

Table S44 (*S*)-AB-FUBINACA percentage remaining (pHHeps)

Time (min)	% parent compound remaining									
	1	2	3	4	5	6	7	8	Average	SD
0	100.0	100.0	100.0	100.0	100.0	100.0	100.0	100.0	100.0	-
3	97.8	91.5	104.5	91.4	92.7	82.6	96.4	89.3	93.3	6.5
6	100.9	98.6	83.5	73.6	65.8	79.8	97.7	88.2	86.0	12.7
9	97.0	89.4	94.0	73.6	68.0	77.1	96.0	88.6	85.5	11.1
15	99.6	95.1	87.2	70.7	78.8	77.1	88.6	86.2	85.4	9.5
30	81.3	71.7	82.7	63.6	68.2	68.5	67.6	76.0	72.4	6.9
45	77.4	79.5	70.8	68.8	63.4	63.2	68.5	64.6	69.5	6.2
60	79.1	67.0	75.1	60.9	62.9	55.4	68.1	64.6	66.6	7.6

Table S45 (*R*)-AB-FUBINACA percentage remaining (pHHeps)

Time (min)	% parent compound remaining									
	1	2	3	4	5	6	7	8	Average	SD
0	100.0	100.0	100.0	100.0	100.0	100.0	100.0	100.0	100.0	-
3	109.7	117.4	118.2	99.0	102.7	60.1	83.7	82.9	96.7	20.0
6	87.6	91.7	84.9	103.8	100.9	65.6	72.0	85.7	86.5	13.0
9	88.1	101.4	79.0	86.1	100.9	63.2	73.0	87.6	84.9	13.1
15	87.0	90.3	87.8	83.6	95.5	67.4	70.6	93.3	84.4	10.2
30	74.1	76.3	85.1	79.7	72.3	59.5	72.0	83.1	75.2	8.0
45	95.9	79.4	84.0	85.3	91.0	66.9	74.2	86.8	82.9	9.2
60	78.4	85.8	84.9	79.8	82.2	70.3	69.0	80.4	78.8	6.2

Table S46 (*S*)-AMB-4en-PICA percentage remaining (pHHeps)

Time (min)	% parent compound remaining									
	1	2	3	4	5	6	7	8	Average	SD
0	100.0	100.0	100.0	100.0	100.0	100.0	100.0	100.0	100.0	-
3	66.1	72.4	81.3	75.5	51.6	63.7	72.0	64.7	68.4	9.0
6	56.1	54.4	70.3	72.3	43.9	45.6	47.1	46.6	54.6	11.2
9	40.9	38.6	43.6	40.6	25.2	27.5	26.7	22.6	33.2	8.5
15	24.2	23.8	27.4	23.8	14.2	15.0	13.1	13.3	19.4	6.0
30	9.1	8.5	9.8	8.3	4.2	4.0	4.4	4.1	6.5	2.6
45	2.4	2.2	3.1	2.6	1.2	1.4	1.3	1.3	1.9	0.7
60	1.0	0.8	1.0	0.7	0.4	0.4	0.4	0.4	0.6	0.3

Table S47 (*R*)-AMB-4en-PICA percentage remaining (pHHeps)

Time (min)	% parent compound remaining									
	1	2	3	4	5	6	7	8	Average	SD
0	100.0	100.0	100.0	100.0	100.0	100.0	100.0	100.0	100.0	-
3	54.5	61.0	78.8	61.7	43.8	52.6	64.3	44.6	57.7	11.4
6	44.2	59.7	63.1	71.0	33.6	33.6	32.8	30.9	46.1	16.1
9	34.2	37.2	33.2	32.1	17.7	18.8	15.8	15.7	25.6	9.3
15	18.9	20.6	18.1	17.2	9.5	9.7	7.6	8.0	13.7	5.5
30	6.8	7.7	6.3	5.8	2.8	2.7	2.6	2.4	4.6	2.2
45	2.1	2.4	2.0	2.0	1.0	1.1	0.9	0.8	1.6	0.7
60	0.9	0.9	0.7	0.8	0.4	0.4	0.3	0.4	0.6	0.3

Table S48 (*S*)-MDMB-FUBINACA percentage remaining (pHHeps)

Time (min)	% parent compound remaining				
	1	2	3	Average	SD
0	100.0	100.0	100.0	100.0	-
3	83.8	88.2	98.9	90.3	7.8
6	64.2	71.6	84.1	73.3	10.1
9	85.7	83.1	71.5	80.1	7.5
15	77.9	74.5	67.5	73.3	5.3
30	41.4	41.8	33.3	38.8	4.8
45	19.1	19.3	13.6	17.3	3.3
60	9.8	9.8	7.7	9.1	1.2

Table S49 (*R*)-MDMB-FUBINACA percentage remaining (pHHeps)

Time (min)	% parent compound remaining				
	1	2	3	Average	SD
0	100.0	100.0	100.0	100.0	-
3	88.5	97.3	93.3	93.0	4.4
6	79.8	85.1	83.7	82.9	2.8
9	82.6	79.9	70.3	77.6	6.5
15	74.6	65.7	70.2	70.2	4.4
30	55.5	48.8	55.0	53.1	3.7
45	60.3	53.4	59.4	57.7	3.7
60	47.9	42.4	32.9	41.1	7.6

Table S50 (*S*)-AB-CHMINACA percentage remaining (pHHeps)

Time (min)	% parent compound remaining									
	1	2	3	4	5	6	7	8	Average	SD
0	100.0	100.0	100.0	100.0	100.0	100.0	100.0	100.0	100.0	-
3	89.1	96.2	93.9	104.9	105.1	89.5	100.2	96.4	96.9	6.2
6	83.7	86.4	86.7	110.1	94.7	78.4	75.6	69.7	85.7	12.5
9	76.1	76.6	78.8	99.1	102.2	79.4	79.0	63.5	81.8	12.7
15	85.9	84.8	97.0	97.9	103.1	91.3	90.6	58.1	88.6	13.8
30	52.8	58.2	55.2	90.2	87.9	78.8	56.9	44.2	65.5	17.5
45	57.1	64.1	64.8	78.8	72.7	59.9	55.1	52.8	63.2	8.9
60	49.4	54.9	52.6	69.6	60.1	57.8	45.2	37.4	53.4	9.8

Table S51 (*R*)-AB-CHMINACA percentage remaining (pHHeps)

Time (min)	% parent compound remaining						
	1	2	3	4	5	Average	SD
0	100.0	100.0	100.0	100.0	100.0	100.0	-
3	94.4	104.3	82.0	77.2	98.4	91.3	11.3
6	90.7	89.3	94.1	73.9	100.9	89.8	10.0
9	85.7	89.3	91.8	78.3	98.1	88.6	7.3
15	75.2	83.6	89.0	83.9	101.3	86.6	9.6
30	62.1	62.0	83.7	68.5	88.6	73.0	12.4
45	75.8	80.7	72.3	62.7	84.3	75.2	8.3
60	65.2	65.9	109.1	67.3	73.4	76.2	18.7

Table S52 (*S*)-AMB-CHMICA percentage remaining (pHHeps)

Time (min)	% parent compound remaining				
	1	2	3	Average	SD
0	100.0	100.0	100.0	100.0	-
3	67.9	62.9	64.2	65.0	2.6
6	57.9	59.2	58.1	58.4	0.7
9	40.6	43.2	46.2	43.3	2.8
15	22.0	24.9	24.3	23.8	1.5
30	8.1	9.3	8.6	8.7	0.6
45	3.3	3.7	3.5	3.5	0.2
60	1.5	1.6	1.5	1.5	0.1

Table S53 (*R*)-AMB-CHMICA percentage remaining (pHHeps)

Time (min)	% parent compound remaining				
	1	2	3	Average	SD
0	100.0	100.0	100.0	100.0	-
3	65.9	64.1	58.1	62.7	4.1
6	61.4	59.5	62.6	61.2	1.5
9	47.5	44.4	43.4	45.1	2.1
15	28.0	25.4	26.8	26.7	1.3
30	12.5	11.5	11.6	11.8	0.6
45	6.1	6.0	5.6	5.9	0.3
60	3.4	3.2	3.0	3.2	0.2

Table S54 (*S*)-5F-AMB-PINACA percentage remaining (pHHeps)

Time (min)	% parent compound remaining				
	1	2	3	Average	SD
0	100.0	100.0	100.0	100.0	-
3	60.0	62.9	57.4	60.1	2.8
6	20.0	22.0	27.5	23.2	3.9
9	8.9	11.5	13.8	11.4	2.4
15	2.6	2.7	3.8	3.0	0.7
30	0.2	0.2	0.4	0.3	0.1
45	0.1	0.1	0.1	0.1	0.0
60	0.5	0.1	0.1	0.2	0.3

Table S55 (*R*)-5F-AMB-PINACA percentage remaining (pHHeps)

Time (min)	% parent compound remaining				
	1	2	3	Average	SD
0	100.0	100.0	100.0	100.0	-
3	70.9	67.9	70.8	69.9	1.7
6	36.2	34.2	36.6	35.6	1.3
9	26.3	26.9	31.5	28.2	2.8
15	11.2	11.3	13.9	12.1	1.5
30	2.5	2.6	4.1	3.1	0.9
45	0.7	0.9	1.1	0.9	0.2
60	0.2	0.1	0.2	0.2	0.1

Table S56 (*S*)-MDMB-4en-PICA percentage remaining (pHHeps)

Time (min)	% parent compound remaining								
	1	2	3	4	5	6	7	Average	SD
0	100.0	100.0	100.0	100.0	100.0	100.0	100.0	100.0	-
3	94.0	82.7	115.2	95.1	83.1	113.0	71.0	93.4	16.3
6	81.5	77.0	104.7	92.1	76.6	79.8	74.0	83.7	11.0
9	88.9	87.4	100.3	79.0	49.1	82.6	75.9	80.4	15.9
15	78.1	77.9	91.5	71.2	41.0	88.4	70.2	74.0	16.6
30	58.1	57.9	60.7	53.2	27.5	61.7	42.3	51.6	12.5
45	37.4	36.5	41.6	33.0	17.5	38.1	35.6	34.2	7.8
60	27.4	27.7	28.5	24.0	12.2	28.5	26.9	25.0	5.9

Table S57 (*R*)-MDMB-4en-PICA percentage remaining (pHHeps)

Time (min)	% parent compound remaining								
	1	2	3	4	5	6	7	Average	SD
0	100.0	100.0	100.0	100.0	100.0	100.0	100.0	100.0	-
3	89.9	87.9	100.4	72.3	88.3	95.6	96.4	90.1	9.1
6	74.0	79.5	85.7	87.4	81.1	77.6	85.2	81.5	4.9
9	80.4	85.6	91.9	79.7	81.3	84.5	82.6	83.7	4.2
15	72.4	78.8	86.2	73.2	72.9	95.0	77.0	79.3	8.4
30	60.2	65.7	64.1	56.3	55.3	70.0	59.4	61.6	5.3
45	43.6	48.2	46.2	39.9	49.2	48.2	49.4	46.4	3.5
60	37.2	40.1	38.8	32.0	43.9	51.2	40.8	40.6	6.0

Table S58 Intrinsic clearance (CL_{int}) with predicted *in vivo* hepatic clearance (CL_H) and hepatic extraction ratio (E_H) for pooled cryopreserved human hepatocyte incubations (n≥3 ±SD) based on a hepatocyte cell density of 99x10^6 hepatocytes per gram liver [10]

Compound	Intrinsic clearance, CL_{int} (mL min^{-1} kg^{-1})	Predicted *in vivo* hepatic clearance, CL_H (mL min^{-1} kg^{-1})	Hepatic extraction ratio, E_H	n
(*S*)-AMB-FUBINACA (**1**)	2975 ±561	15.21 ±0.88	0.72 ±0.042	8
(*R*)-AMB-FUBINACA (**1**)	2366 ±429	-	-	8
(*S*)-5F-AMB-PINACA (**10**)	2225 ±115	18.06 ±0.13	0.86 ±0.006	3
(*R*)-5F-AMB-PINACA (**10**)	1501 ±80.5	-	-	3
(*S*)-AMB-4en-PICA (**7**)	1116 ±336	15.25 ±1.28	0.73 ±0.061	8
(*R*)-AMB-4en-PICA (**7**)	1470 ±570	16.53 ±1.39	0.79 ±0.066	8
(*S*)-AMB-CHMICA (**9**)	935 ±44.8	7.31 ±0.23	0.35 ±0.011	3
(*R*)-AMB-CHMICA (**9**)	849 ±35.3	-	-	3
(*S*)-MDMB-4en-PINACA (**5**)	632 ±147	4.82 ±0.85	0.23 ±0.041	7
(*R*)-MDMB-4en-PINACA (**5**)	335 ±61.6	4.86 ±0.67	0.23 ±0.032	7
(*S*)-5F-MDMB-PINACA (**2**)	559 ±125	7.67 ±1.13	0.37 ±0.054	12
(*R*)-5F-MDMB-PINACA (**2**)	309 ±23.6	7.78 ±0.38	0.37 ±0.018	6
(*S*)-4F-MDMB-BINACA (**4**)	432 ±20.0	11.68 ±0.24	0.56 ±0.011	3
(*R*)-4F-MDMB-BINACA (**4**)	128 ±34.6	8.38 ±1.40	0.40 ±0.067	4
(*S*)-MDMB-FUBINACA (**6**)	275 ±58.0	1.29 ±0.25	0.06 ±0.012	3
(*R*)-MDMB-FUBINACA (**6**)	221 ±37.7	-	-	3
(*S*)-MDMB-4en-PICA (**12**)	197 ±38.4	5.15 ±0.75	0.25 ±0.036	7
(*R*)-MDMB-4en-PICA (**12**)	162 ±22.16	6.08 ±0.60	0.29 ±0.028	7
(*S*)-5F-MDMB-PICA (**3**)	188 ±21.5	7.49 ±0.56	0.36 ±0.027	3
(*R*)-5F-MDMB-PICA (**3**)	134 ±22.3	5.94 ±0.72	0.28 ±0.034	3
(*S*)-AB-CHMINACA (**11**)	194 ±67.5	4.26 ±1.08	0.20 ±0.051	6
(*R*)-AB-CHMINACA (**11**)	197 ±50.3	-	-	5
(*S*)-AB-FUBINACA (**8**)	101 ±32.0	1.92 ±0.55	0.09 ±0.026	8
(*R*)-AB-FUBINACA (**8**)	105 ±14.6	-	-	8

Reference

10. Barter, Z.E.; Bayliss, M.K.; Beaune, P.H.; Boobis, A.R.; Carlile, D.J.; Edwards, R.J.; Houston, J.B.; Lake, B.G.; Lipscomb, J.C.; Pelkonen, O.R.; Tucker, G.T.; Rostami-Hodjegan, A. Scaling factors for the extrapolation of in vivo metabolic drug clearance from in vitro data: reaching a consensus on values of human microsomal protein and hepatocellularity per gram of liver. *Curr. Drug. Metab.* **2007**, *8*, 33-45. doi: 10.2174/138920007779315053.
